# Changes in Reading Attitudes and Attitudes Toward Book-Reading Habits Following Literature Circles Among Preservice Turkish Language Teachers: An Embedded Mixed-Methods Study

**DOI:** 10.3390/bs16101757

**Published:** 2026-09-27

**Authors:** Yasemin Baki, Gökhan Kahveci

**Affiliations:** 1 Department of Turkish and Social Sciences, Faculty of Education, Recep Tayyip Erdoğan University, 53200 Rize, Türkiye; yasemin.baki@erdogan.edu.tr; 2 Department of Educational Sciences, Faculty of Education, Recep Tayyip Erdoğan University, 53200 Rize, Türkiye

**Keywords:** literature circles, reading attitude, attitudes toward book-reading habits, preservice Turkish language teachers, embedded mixed methods

## Abstract

Reading attitudes and attitudes toward book-reading habits are related but distinct constructs. This study examined within-group pretest–posttest differences in these constructs and participants’ experiences of a seven-week literature-circle program. A quantitatively driven embedded mixed-methods design combined a single-group pretest–posttest phase (*n* = 36) with post-program interviews (*n* = 35) at a public university in Türkiye. Analyses included paired-samples *t* tests, effect sizes, 95% confidence intervals, Holm adjustment, and Wilcoxon signed-rank sensitivity analyses. Interviews underwent hybrid deductive–inductive content analysis, and findings were integrated through joint displays. After Holm adjustment, the reading-attitude outcome meeting the significance threshold depended on the method: perceived importance and value met the threshold in the *t* test (*d*_z_ = 0.54), whereas overall reading attitude did so in the Wilcoxon analysis. No outcome met the threshold in both analyses. Estimates for contribution to personal and social development and all outcomes concerning attitudes toward book-reading habits were small and imprecise, with confidence intervals including zero. Participants reported interest, value awareness, purposeful reading, regular reading during the program, and peer sharing. These retrospective accounts contextualized rather than independently confirmed the quantitative findings. The results do not indicate uniform improvement across outcomes, and the single-group design precludes causal attribution.

## 1. Introduction

Reading is a multidimensional process that extends beyond recognizing written words to constructing meaning from text, connecting new information with prior knowledge, drawing inferences, and evaluating what is read in light of personal experience. Exposure to print and independent reading experiences are associated with vocabulary knowledge, reading comprehension, and academic development from early childhood through adulthood (Mol & Bus, 2011). Nevertheless, possessing reading skills alone may not be sufficient for reading to become a willing, regular, and sustained practice. The time individuals devote to reading, the variety of texts they read, the value they attach to reading, and their willingness to continue reading may also shape the quality of their reading experience.

The amount of reading and the variety of texts read are associated with different dimensions of reading motivation (Wigfield & Guthrie, 1997). However, the relationships among reading motivation, reading activity, and reading achievement are not equally strong across all dimensions (Baker & Wigfield, 1999). The differing relationships of intrinsic and extrinsic reading motivation with reading behavior and reading competence further indicate that the reading process cannot be explained by a single affective variable (Schiefele et al., 2012). Reading experiences should therefore be evaluated by distinguishing among related but conceptually different constructs, such as reading ability, motivation, attitude, and behavior.

Reading attitude, one such construct, refers to an individual’s feelings, thoughts, and evaluations concerning reading. Students may hold different attitudes toward academic and recreational reading, suggesting that reading attitude can vary across reading contexts (McKenna et al., 1995). Enjoyment of reading and confidence in one’s reading ability are also among the affective factors that should be considered when examining students’ reading experiences (Sainsbury & Schagen, 2004). Although reading attitude is significantly associated with reading achievement, the strength of this relationship may vary according to factors such as age and educational level (Petscher, 2010). Reading attitude and reading achievement have also been reported to display a reciprocal pattern over time (Kush et al., 2005). Among students in the early elementary grades, the relationships among reading motivation, reading amount, and reading comprehension have been shown to differ across grades and analytical models (Stutz et al., 2016). In another longitudinal study of early elementary students, reading amount did not mediate the association between intrinsic reading motivation and later reading comprehension, although evidence emerged for partial mediation in the reverse direction (Soemer & Schiefele, 2018). These findings suggest that the relationships among reading motivation, reading behavior, and reading comprehension may be directionally complex.

Although reading attitude and attitudes toward book-reading habits are related, they do not represent the same construct. Reading attitude concerns an individual’s affective and cognitive orientation toward reading, whereas attitudes toward book-reading habits concern how individuals evaluate the regularity, meaning, indispensability, and development of book reading (Sarar Kuzu & Doğan, 2015; Susar Kırmızı, 2012). A favorable attitude toward reading may support regular reading, but it does not necessarily indicate that an individual reads books regularly. Similarly, reading regularly during a specific program does not demonstrate that the behavior will continue after the program has ended. The instrument used in the present study measures neither reading frequency nor sustained reading behavior directly; instead, it assesses preservice teachers’ attitudes toward book-reading habits. Accordingly, the second quantitative variable in this study is not “book-reading habits” but “attitudes toward book-reading habits.” This distinction is important for interpreting the findings and defining the scope of the observed pretest–posttest differences.

Instructional practices used during reading may shape the quality of students’ engagement with texts. Practices that encourage students not merely to read and finish a text but also to reflect on it, ask questions, develop their own interpretations, and share their responses with others can make reading a more participatory experience. Instructional practices that support student interest, choice, collaboration, and meaning-making have been associated with reading comprehension and engagement (Guthrie et al., 2004). Research examining reading instruction, motivation, engagement, and achievement together also suggests that classroom reading practices should be considered alongside students’ affective and cognitive characteristics (Guthrie et al., 2013).

Literature circles are one instructional practice that brings together the individual and social dimensions of reading. In literature circles, students read assigned or self-selected texts in small groups and share the observations, questions, and connections they have prepared in accordance with their roles. Participating in text selection, task allocation, and discussions allows students to assume a degree of responsibility and autonomy during reading (Blum et al., 2002). Reading thus becomes not merely an individual task to be completed but a social process of meaning-making in which different interpretations are compared. The purpose of literature circles, however, is not limited to assigning roles or encouraging students to discuss the same text. Establishing a supportive discussion environment in which participants can express their ideas comfortably, together with teacher facilitation when needed, is important for the effective implementation of literature circles (Clarke & Holwadel, 2007).

In this study, the possible relationship between literature circles and reading outcomes was considered through a conceptual distinction between outcomes that are relatively close to the immediate program experience and broader attitudinal orientations that develop over time. Opportunities to contribute to book selection may support a sense of autonomy, preparation for assigned roles may foster responsibility, and peer discussions may allow participants to share different interpretations of what they read. These features may be more directly related to interest in and enjoyment of reading and to perceptions of reading’s importance and value (Blum et al., 2002; Gambrell, 2011; Guthrie et al., 2004, 2013). By contrast, evaluations of reading’s contribution to personal and social development reflect a broader attitudinal domain, while attitudes toward book-reading habits encompass more enduring orientations concerning regularity, meaning, indispensability, and habit development. Literature-circle components were therefore not assumed to relate equally closely to every attitude dimension or to be accompanied by similar pretest–posttest patterns across all outcomes. This framework was used to explain why the outcome domains needed to be examined separately, rather than to test a causal pathway.

Students who have participated in literature circles have identified talking about books, listening to peers, and expressing their ideas about what they read as salient features of the practice (Certo et al., 2010). Group discussions have also been reported to provide opportunities not only to understand texts but also to reflect on how students speak and listen during these discussions (Mills & Jennings, 2011). These findings suggest that literature circles may offer opportunities to listen to peers’ perspectives, compare different interpretations, and reconsider one’s own ideas alongside individual engagement with a text. Such outcomes should nevertheless be evaluated in relation to the quality of the discussions, group interaction, text selection, participant preparation, and teacher guidance.

Literature circles have been examined across different age groups, language-learning contexts, and implementation settings. In a study involving learners of English, literature circles were evaluated in relation to reading comprehension and classroom peer support (McElvain, 2010). The relationship of online literature circles with literary text comprehension and self-regulated language-learning skills has also been investigated (Kassem, 2022). Studies in which literature circles were redesigned to incorporate multimodal student responses found that students could respond to literary texts through different modes of representation (Cloonan et al., 2020). Applications in foreign-language education have shown that literature circles can incorporate student roles, peer interaction, and teacher guidance (Shelton-Strong, 2012). Literature circles have also been adapted to online instruction and implemented in online discussion environments (Whittingham, 2013). Other studies have examined reading comprehension and motivation (Meredith, 2015), reading motivation and skill (Gailea et al., 2022), and students’ perceptions of reading circles using local cultural narratives (Djehatu et al., 2026). Wiki-supported literature circles have also been examined in relation to learners’ online self-regulation and attitudes toward collaborative learning (Su et al., 2019), as well as social regulation strategies, learning engagement, and learning outcomes (Li et al., 2021). Although these studies indicate that literature circles can be adapted to different contexts, each focuses on different participant groups, learning objectives, and outcome variables.

A recent meta-analysis synthesizing research on second-language reading comprehension found that literature circles were associated with favorable reading-comprehension outcomes, although the results varied according to factors such as students’ educational level and the assessment instruments used (Ma, 2025). Although this finding provides important evidence regarding the relationship between literature circles and reading comprehension, it does not constitute direct evidence concerning general reading attitudes or attitudes toward book-reading habits. A recent higher-education study also examined academic reading circles in relation to students’ perceptions of the practice, motivation, and academic reading outcomes (Ament et al., 2025). The cognitive outcomes of literature circles, participants’ evaluations of the practice, and general reading attitudes should therefore not be treated as interchangeable.

For preservice teachers, reading attitudes and attitudes toward book-reading habits have implications beyond individual academic development. Teachers’ own reading experiences may be related to the reading environments they create in their classrooms and the reading activities they offer their students. Some preservice and in-service teachers have been reported to show limited interest in personal reading (Nathanson et al., 2008). A large-sample study of university students likewise noted that some students preparing for the teaching profession may demonstrate limited willingness and commitment to reading (Applegate et al., 2014). Supporting teachers’ development as readers is important for creating classroom communities that value reading for pleasure (Cremin et al., 2009). Differences have been identified in the classroom reading practices of teachers who read in their personal lives (Morrison et al., 1998), and the relationship between teachers’ personal reading habits and instructional practices has also been examined elsewhere (McKool & Gespass, 2009). These findings suggest that teacher education programs should attend not only to preservice teachers’ knowledge of reading instruction but also to their identities as readers.

This need is particularly salient for preservice Turkish language teachers. Turkish language teachers are expected to develop their students’ reading skills, introduce them to a range of text genres, and foster a positive reading culture. Preservice teachers’ own reading attitudes, attitudes toward book-reading habits, and identities as readers therefore constitute an important part of teacher education. Preservice Turkish language teachers’ attitudes toward reading culture have been found to vary according to several personal and educational characteristics (Baki & Gökçe, 2020). Their reading experiences should consequently be considered not only in relation to individual academic competence but also in terms of the classroom reading environments they may create in their future practice.

Studies conducted with preservice teachers in Türkiye show that literature circles have previously been examined in teacher education in relation to various outcomes. Preservice Turkish language teachers have evaluated literature circles favorably in terms of collaboration, recognizing different perspectives, expressing their ideas, and examining texts in greater depth (Aytan, 2018). Literature circles have also been investigated in relation to preservice teachers’ reading-comprehension levels (K. Yıldırım et al., 2019). Doğan et al. (2020) used mixed methods to examine the reading attitudes of 21 preservice teachers who participated in literature circles for an academic year. Serin and Turan (2022), meanwhile, employed a controlled quasi-experimental design in a nine-week program to investigate preservice Turkish language teachers’ attitudes toward book-reading habits and their views of the program. Other teacher-education studies have examined literature circles in relation to text-analysis skills and the desire to read among prospective Turkish teachers (Karatay, 2017), the preparation of preservice teachers as agents of social justice in a reading methods course (Davis & Bush, 2021), and the implementation of equity literature circles with preservice teachers (Robertson & Sivia, 2025).

Recent research has also linked literature circles to other reading-related constructs and skills. In one study involving preservice Turkish language teachers, the practice was examined in relation to reading culture, planned reading, in-depth and inferential comprehension, and the comprehension and interpretation of visual materials (Baki, 2023). Another study evaluated outcomes associated with curiosity and exploration, creative reading, and visual literacy (Baki, 2025). A study involving fifth-grade students examined literature circles in conjunction with reading-comprehension strategies, reading attitudes, and reading habits (Çağ, 2024). A descriptive content analysis of research on literature circles in Turkish language education further indicated that studies have addressed different educational levels and a variety of reading skills (Tavşanlı & Ulaş, 2023). A mixed-methods study with primary school students also examined reading fluency, reading comprehension, and reader responses (Kaya Tosun & Doğan, 2020).

Previous research has examined literature circles in relation to preservice teachers’ reading attitudes, attitudes toward book-reading habits, and various reading experiences (Baki, 2023, 2025; Doğan et al., 2020; Serin & Turan, 2022). Accordingly, the contribution of the present study does not rest on applying literature circles to an entirely new participant group or introducing an outcome that has not previously been examined. Instead, the study examines whether general affective and cognitive evaluations of reading, attitudes concerning the meaning and indispensability of regular book reading, and reported reading practices during the program reflect the same underlying response. This comparison makes it possible to examine whether viewing reading as interesting and valuable during a short, externally structured program necessarily follows the same pattern as viewing regular book reading as a sustainable everyday practice. Favorable program experiences, general reading attitude, attitudes toward book-reading habits, and program-bound reading routines are therefore not treated as interchangeable indicators. Considering quantitative score patterns alongside experiences such as following a weekly reading schedule, using role sheets, engaging closely with texts, and discussing texts with peers helps distinguish findings that concern overlapping constructs from those that are related but not equivalent. The intended theoretical and methodological contribution therefore lies not simply in administering two attitude instruments to the same group, but in interpreting relationships among distinct reading-related constructs with explicit attention to construct scope, data type, and the timing of data collection.

The purpose of this study was to examine within-group pretest–posttest differences in the reading attitudes and attitudes toward book-reading habits of preservice Turkish language teachers who participated in a seven-week literature-circle program and to explore participants’ post-program accounts of their reading experiences. The relationship between the quantitative and qualitative findings was considered in light of the scope of the constructs examined and the timing of data collection. The study addressed the following research questions:

1. What pretest–posttest differences are observed in preservice Turkish language teachers’ overall and subscale reading-attitude scores?
2. What pretest–posttest differences are observed in preservice Turkish language teachers’ overall and subscale scores for attitudes toward book-reading habits?
3. How do preservice Turkish language teachers describe their reading experiences during the literature-circle program and the ways in which they perceive these experiences to relate to their reading attitudes and attitudes toward book-reading habits?
4. How are the quantitative score patterns and qualitative accounts concerning reading attitudes and attitudes toward book-reading habits related when the scope of the constructs and the timing of data collection are taken into account?

## 2. Materials and Methods

### 2.1. Research Design

This study employed a mixed-methods approach in which quantitative and qualitative data were considered together within the same research problem (Creswell, 2014; Creswell & Plano Clark, 2011/2014). An intervention-based embedded mixed-methods design that prioritized the quantitative component was adopted. This design allowed the quantitative findings obtained during the program to be considered alongside qualitative data concerning participants’ experiences. The design can be represented as QUAN (pretest–program–posttest) → qual, with the qualitative phase embedded in the evaluation of the program to contextualize and explain the quantitatively prioritized pretest–posttest findings.

In the quantitative component, differences in preservice Turkish language teachers’ reading attitude scores and scores for attitudes toward book-reading habits before and after the program were examined using a single-group pretest–posttest design. Because the study included neither a control group nor random assignment, the quantitative component was intended to compare participants’ pretest and posttest scores rather than test the causal effects of literature circles. In the qualitative component, semi-structured interviews were conducted after the program to explore how participants experienced the literature circle process and how this experience was reflected in their views of reading. This component was treated not as a separate case study but as a qualitative phase embedded within the evaluation of the program.

After the quantitative and qualitative data had been analyzed separately using their respective methods, the scale dimensions and the themes derived from the interviews were brought together in joint displays. The integration process examined where the two types of data converged and diverged. This approach allowed participants’ experiences of the program to be incorporated into the study findings alongside the pretest–posttest comparisons (Fetters et al., 2013).

### 2.2. Participants

Thirty-six preservice teachers enrolled in a Turkish Language Teaching Program at a public university in northern Türkiye participated in the study. Convenience sampling was used because the participants were enrolled in the course during the study period and were accessible throughout the 12-week research process. The study group comprised 28 women (77.8%) and eight men (22.2%). All 36 participants in the quantitative phase were invited to participate in the post-program semi-structured interviews. One participant declined, and qualitative data were therefore obtained from 35 preservice teachers, including 27 women and eight men. Thus, no separate sample was recruited for the qualitative phase; the interviews were conducted with participants who had completed the program and agreed to be interviewed. Participation was voluntary. Participants were between 19 and 21 years of age and were enrolled in the second year of the undergraduate Turkish language teaching program. None of the participants had prior experience with literature circles, and they were unfamiliar with the books selected for the program. Data on participants’ usual reading frequency before the program were not collected. Participants were informed about the purpose of the study and the program procedures and were assured that their decision whether to participate would have no bearing on their academic performance or course grades. The present study involved a different participant cohort, was implemented in a different academic semester, used different books, and generated quantitative data and qualitative interviews that were entirely separate from those reported in Baki (2023, 2025). Accordingly, there was no overlap between the studies in participants, implementation period, selected books, interviews, or datasets. References to Baki (2022, 2023) in the description of the intervention indicate only that these studies informed the orientation content; they do not indicate that participants or data from the earlier studies were reused.

### 2.3. Data Collection Instruments

Quantitative data were collected using the Preservice Teachers’ Attitudes toward Book-Reading Habits Scale and the Reading Attitude Scale for Preservice Teachers. A personal information form was used to obtain participants’ background information, while a semi-structured interview form was used to explore their experiences of the program.

#### 2.3.1. Preservice Teachers’ Attitudes Toward Book-Reading Habits Scale

The Preservice Teachers’ Attitudes toward Book-Reading Habits Scale, developed by Susar Kırmızı (2012), was used to assess participants’ attitudes toward book-reading habits. This five-point Likert-type scale consists of 34 items across three dimensions. The “meeting learning needs and enjoyment” dimension comprises 22 items, the “meaning and indispensability of book-reading habits” dimension comprises eight items, and the “development of book-reading habits” dimension comprises four items. Possible scores for these dimensions range from 22 to 110, 8 to 40, and 4 to 20, respectively. Total scores range from 34 to 170, with higher scores indicating more favorable attitudes toward book-reading habits. Negatively worded items were reverse-scored in accordance with the scale’s scoring key. The subscale and total scores were calculated using the item order and dimensional structure of the administered final form. The total score was calculated as the sum of the three dimension scores. In the original scale-development study, Cronbach’s alpha coefficients were reported as 0.78 for meeting learning needs and enjoyment, 0.88 for the meaning and indispensability of book-reading habits, 0.72 for the development of book-reading habits, and 0.95 for the total scale (Susar Kırmızı, 2012). In the present study, the corresponding Cronbach’s alpha coefficients at pretest were 0.830, 0.730, and 0.762 for the three dimensions and 0.901 for the total score. At posttest, the coefficients were 0.797, 0.720, and 0.765 for the dimensions and 0.894 for the total score. These coefficients indicated acceptable internal consistency for the total and subscale scores in the present sample.

#### 2.3.2. Reading Attitude Scale for Preservice Teachers

The Reading Attitude Scale for Preservice Teachers, developed by Sarar Kuzu and Doğan (2015), was used to assess participants’ attitudes toward reading. This five-point Likert-type scale consists of 38 items across three dimensions. The “contribution to personal and social development” dimension comprises 19 items, the “interest in and enjoyment of reading” dimension comprises 14 items, and the “perceived importance and value of reading” dimension comprises five items. Possible scores for these dimensions range from 19 to 95, 14 to 70, and 5 to 25, respectively. Total scores range from 38 to 190, with higher scores indicating more favorable attitudes toward reading. The scale contains 30 positively worded and eight negatively worded items. The negatively worded items were reverse-scored in accordance with the scale’s scoring key. The subscale and total scores were calculated using the item order and dimensional structure of the administered final form. The total score was calculated as the sum of the three dimension scores. In the original scale-development study, Cronbach’s alpha coefficients were reported as 0.922 for contribution to personal and social development, 0.853 for interest in and enjoyment of reading, 0.654 for perceived importance and value of reading, and 0.948 for the total scale (Sarar Kuzu & Doğan, 2015). In the present study, the corresponding Cronbach’s alpha coefficients at pretest were 0.834, 0.795, and 0.853 for the three dimensions and 0.875 for the total score. At posttest, the coefficients were 0.918, 0.725, and 0.695 for the dimensions and 0.826 for the total score. The coefficients ranged from 0.695 to 0.918 across the two administrations. The dimension-level findings, particularly those for the five-item perceived importance and value dimension at posttest, were interpreted with consideration of these reliability estimates.

#### 2.3.3. Semi-Structured Interview Form

A semi-structured interview form developed by the researchers was used to explore how participants experienced the literature circle process and how this process was reflected in their views of reading. Semi-structured interviews allow discussions to be organized around a common set of topics while giving participants opportunities to elaborate on their experiences (Cohen et al., 2011; Patton, 2002). The interview form was developed with explicit consideration of the study purpose, the relevant literature, and the constructs assessed by the two quantitative instruments. Question 1 addressed the overall construct of reading attitude, while Questions 2–4 were aligned with the three dimensions of the Reading Attitude Scale for Preservice Teachers. Questions 6–8 were informed by the three dimensions of the Preservice Teachers’ Attitudes toward Book-Reading Habits Scale. Question 5 elicited participants’ retrospective comparisons of their reading practices before and during the program. This question was not treated as an objective measure of reading behavior or as a direct qualitative counterpart of the attitude scale. Questions 9–11 focused on the roles undertaken by participants, the different stages of implementation, and the positive, negative, facilitating, and challenging aspects of the process.

This planned relationship between the interview questions and the scale constructs enabled conceptually related quantitative and qualitative evidence to be compared. However, the qualitative findings were not treated as a source of confirmation independent of the quantitative results. The conceptual domains used when developing the questions were not applied as closed coding categories; data-driven codes were permitted so that experiences outside these domains could be retained in the analysis. The original Turkish wording of the complete interview guide, its English translation, and the conceptual basis of each question are provided in Supplementary Table S1.

The draft form was reviewed by two subject-area experts for its alignment with the purpose of the study, clarity, and scope. Based on their feedback, the form was pilot-tested with five preservice teachers who were not included in the main study group. The pilot test indicated that the questions were clear, after which the form was finalized. Following completion of the program, individual face-to-face interviews were conducted with the 35 preservice teachers who agreed to participate. The interviews lasted approximately 40 min on average.

#### 2.3.4. Personal Information Form

The personal information form developed by the researchers was used to collect categorical background information about the preservice teachers.

### 2.4. Data Collection Procedure

In an embedded mixed-methods design, quantitative and qualitative data may be collected either sequentially or concurrently (Creswell & Plano Clark, 2011/2014). In the present study, quantitative data were collected by administering the scales as pretests and posttests, while qualitative data were obtained through semi-structured interviews conducted after the program. With one week allocated to each book, the literature circle program lasted seven weeks. Including the orientation training, pretest and posttest administration, and semi-structured interviews, the full research process was completed over 12 weeks. The chronological sequence of the 12-week research process, from pretest administration through the semi-structured interviews, is shown in Figure 1.

Before the program, the researcher provided the preservice teachers with orientation training on literature circles. The training was spread over one week and two additional days and totaled eight hours. It covered the characteristics of literature circles, the preparation and use of role sheets, program procedures, meeting rules, and the products to be prepared during the program. Participants also watched sample implementation videos, were introduced to the virtual classroom created in Google Classroom, and agreed on meeting days and times. The training content drew on the researcher’s previous studies (Baki, 2022, 2023).

The researcher developed the role sheets used in the program by drawing on examples from the literature (Avcı et al., 2013; Doğan et al., 2019). Nine roles were defined, comprising four core roles and five optional roles. The core roles were Connector, Questioner, Passage Expert, and Illustrator. The optional-role pool comprised Summarizer, Word Detective, Setting Tracker, Character Expert, and Observer.

The Connector established links between the book and other texts, authors, genres, or experiences from everyday life. The Questioner prepared questions intended to initiate and deepen the discussion and facilitated the group discussion. The Passage Expert selected important or noteworthy passages from the text and explained why they were significant. The Illustrator represented a scene, symbol, expression, or passage selected by the group in visual form. The Summarizer prepared a concise summary of the book, while the Word Detective identified unfamiliar, notable, or important words and explained their meanings. The Setting Tracker examined and described the settings in which the events took place, and the Character Expert considered the characteristics of the characters in the book. The Observer helped coordinate the date and time of the meeting, made the required video recording, documented the group members’ preparation and participation in the discussion on the observer form, and monitored the uploading of the weekly materials to the virtual classroom.

Although the Observer was included in the optional-role pool within the general role framework, this role was required in every group in the present study. At each weekly meeting, the four core roles and the Observer role were assigned, while the sixth role was selected from the remaining optional roles in accordance with participants’ preferences. Roles were rotated weekly, and each participant assumed each of the four core roles at least once during the seven-week program. Detailed role descriptions, blank role sheets, and the blank observer form are provided in Supplementary File S2.

Allowing participants to take part in book selection is regarded as one way of supporting interest and engagement in literature circles (Moeller & Moeller, 2007). Before the program began, approximately 50 preservice teachers, including all 36 study participants, were contacted through existing WhatsApp groups and invited to nominate, without being given a predetermined list, books they wished to read. The nominations were tallied, and the seven titles receiving the highest numbers of votes were selected for the program. Once the list had been finalized, the 36 study participants were asked directly whether they had previously read any of the selected books. None reported having read any of the seven titles before the program. The selected works shared the broad characteristic of being written for children or young readers. However, they were not formally matched in terms of genre, length, thematic content, conceptual demands, or intended readership. One week was allocated to each title, and all groups read the same seven books during the seven-week program. Descriptive characteristics of the books are presented in Table 1.

After the books had been selected, the 36 participants voluntarily formed six groups of six. During the orientation week, the researcher met separately with each group to explain the role-allocation process, and the groups selected their own names. After the groups had been formed, the days and times of the weekly meetings were scheduled. Before the main implementation began, a trial literature-circle session was conducted using a short text. The process was reviewed after the trial, and any aspects that participants had difficulty understanding were explained again. Each of the six groups met once per week throughout the seven-week program, resulting in the completion of all 42 planned group meetings. Following each week’s reading, the groups met to discuss the book. The meetings lasted approximately 60 min, with some variation depending on the flow of the group discussion. Meetings were generally held on the faculty premises, although groups could use another location when meeting there was not feasible. After the trial session, the weekly meetings were student-led, and the participant assigned the Questioner role facilitated the discussion. The researcher did not lead the discussions but periodically attended portions of the meetings for brief periods to monitor the process, particularly the implementation of the Observer role.

A video excerpt of approximately 10–15 min was recorded from each meeting and uploaded to the virtual classroom to document the implementation process. Because of storage limitations, the meetings were not recorded in full. Each participant’s completed role sheet and the group’s observer form were uploaded weekly to Google Classroom and reviewed by the researcher. All groups completed the planned meetings. In the few instances of illness-related absence, the participants concerned viewed the recorded excerpt and uploaded their completed role sheets to the virtual classroom. No major departures from the planned procedure were documented during the weekly monitoring. At the end of each weekly discussion, each group prepared a poster about the book. Although participants had been introduced to different product formats during the orientation, posters were the only products prepared as part of the present study. The role sheets and posters were reviewed by the researcher and shared through the virtual classroom. These materials were not graded and did not contribute to participants’ course grades or academic assessment. Upon completion of the seven-week literature-circle program, the Reading Attitude Scale for Preservice Teachers and the Preservice Teachers’ Attitudes toward Book-Reading Habits Scale, both of which had been administered at pretest, were readministered as posttests. Semi-structured interviews were conducted during the following week and lasted approximately 40 min on average. The semi-structured interviews were conducted in Turkish, the participants’ native language, audio-recorded, and transcribed verbatim. The interviews were conducted by one of the researchers, who was also the participants’ course instructor. Informed consent was obtained from all participants. After the program, all 36 participants were invited to take part in the interviews. Interview participation was also voluntary, and no qualitative data were collected from the one participant who declined. Participants were explicitly informed that their decision whether or not to participate in the study or the interview would have no effect on their academic performance or course grades. Quotations were reported using participant codes rather than personal names, and potentially identifiable raw data were not made publicly available in order to protect participant privacy.

### 2.5. Data Analysis

The quantitative and qualitative data were analyzed sequentially. The quantitative data obtained from the scales were analyzed first, followed by the qualitative data obtained from the semi-structured interviews. This sequence allowed the quantitative findings to be considered alongside participants’ accounts (Creswell, 2014; Johnson & Christensen, 2004).

#### 2.5.1. Quantitative Data Analysis

Data obtained from the pretest and posttest administrations of the Reading Attitude Scale for Preservice Teachers and the Preservice Teachers’ Attitudes toward Book-Reading Habits Scale were entered into IBM SPSS Statistics (version 27; IBM Corp., Armonk, NY, USA) and analyzed using the software (Kalaycı, 2018); data cleaning was conducted before the analyses (Tabachnick & Fidell, 2007). Frequency analyses confirmed that all item responses fell within the permissible response ranges of the instruments. Negatively worded items were reverse-scored in accordance with the scoring keys of the respective instruments. Subscale and total scores were calculated using the item order and dimensional structure of the administered final forms. For each instrument, the total score was calculated as the sum of its three-dimension scores. All 36 participants had complete item-level data for both instruments at both measurement points; therefore, no missing-data imputation was required.

The pretest–posttest comparisons were conducted using paired scores from 36 participants. Change scores were calculated by subtracting pretest scores from posttest scores; therefore, positive values indicated an increase at posttest. The distributions of the change scores for the eight outcomes were examined using skewness, excess kurtosis, Shapiro–Wilk tests, and boxplots (Chou & Bentler, 1995). Potential outliers in the boxplots were flagged using the 1.5 interquartile-range criterion, and change scores with absolute standardized z scores greater than 3.29 were treated as extreme values. Flagged observations were checked against the item-level responses and the permissible score ranges of the instruments. Because no data-entry or scoring errors were identified, no observations were excluded from the analyses. To describe possible floor and ceiling effects, the observed scores at each measurement point were compared with the theoretical minimum and maximum values of the respective scales.

Mean changes were examined using two-sided paired-samples *t* tests with an alpha level of 0.05. Regardless of statistical significance, the mean change, its 95% confidence interval, and Cohen’s *d*_z_ were reported for every comparison. Cohen’s *d*_z_ was calculated by dividing the mean change by the standard deviation of the change scores (Lakens, 2013). The two total-score comparisons and six dimension-level comparisons across the two scales yielded eight comparisons. These comparisons were treated as a single family, and the Holm procedure was applied to control the familywise Type I error rate (Holm, 1979).

Two-sided Wilcoxon signed-rank tests were also conducted as sensitivity analyses to assess whether the inferences were sensitive to the analytic method. The Holm procedure was applied to the eight Wilcoxon comparisons as a separate family from the paired-samples *t* tests. Both unadjusted and Holm-adjusted *p* values are reported for the two analytic approaches. The 95% confidence intervals for Cohen’s *d*_z_ were estimated by inverting the noncentral t distribution. All reported confidence intervals were pointwise intervals and were not adjusted for multiple comparisons or for possible dependence among participants within the same literature-circle group.

Because the available course cohort determined the sample size, no a priori sample-size calculation was conducted. Under the assumptions of a two-sided paired-samples *t* test with α = 0.05 and independent paired observations, a sample of 36 provided 80% power to detect an effect of approximately *d*_z_ = 0.48 or larger. This calculation describes the sensitivity of the design rather than retrospective power associated with the observed effects (Lakens, 2022).

#### 2.5.2. Qualitative Data Analysis

Qualitative data obtained from the semi-structured interviews were examined using a hybrid qualitative content analysis that combined deductive and inductive coding (Fereday & Muir-Cochrane, 2006; Hsieh & Shannon, 2005; A. Yıldırım & Şimşek, 2013). The analysis was conducted using the complete interview transcripts of the 35 preservice teachers who participated in the qualitative phase. Before coding began, the researchers read the transcripts several times to familiarize themselves with the participants’ accounts and the interview data as a whole.

The initial analytical framework was informed by the research questions, the relevant literature, and the six dimensions of the two scales used in the study. These dimensions were treated as provisional conceptual domains that guided the initial examination of the data, rather than as predetermined codes or closed categories into which all statements had to be placed. Accordingly, no fixed list of initial codes or predetermined number of codes was established before coding. The six scale dimensions provided the deductive starting framework, whereas more specific codes were generated inductively through close examination of the transcripts. This approach was intended to preserve experiences that did not correspond directly to the scale dimensions.

The coding unit was defined as a meaning unit consisting of a sentence or a set of adjacent sentences expressing a coherent idea or experience. Each transcript was examined in full, and a meaning unit could be assigned more than one code when it addressed multiple aspects of the participant’s experience. Data-driven codes were created for statements that were not adequately represented by the provisional conceptual domains. Experiences involving close and strategic reading, the use of role sheets, group interaction, self-expression, and regular reading during the program were therefore retained even when they did not correspond directly to the constructs assessed by the scales. The qualitative themes and categories were not required to reproduce the scale dimensions.

As coding progressed, codes with overlapping meanings were combined, overly broad codes were divided, and codes that were not adequately supported by the data were reconsidered. A codebook was developed containing code labels, definitions, inclusion and exclusion criteria, and illustrative extracts. Related codes were organized into categories, and patterns across the categories were used to develop the themes. The final analytical structure comprised three themes and eight categories concerning reading attitudes and three themes and nine categories concerning attitudes toward book-reading habits. After the coding and thematic structure had been developed, the complete set of transcripts was reviewed again. Particular attention was given to accounts that did not fit, qualified, or reflected less favorable experiences than the predominant patterns.

To strengthen the consistency and transparency of the analytical interpretations, the two researchers and a subject-area expert independently reviewed all 35 interview transcripts and the resulting coding structure without access to one another’s assessments. This review was intended to subject the code definitions and analytical interpretations to independent scrutiny rather than to treat qualitative coding as a mechanical measurement procedure or to present a statistical coefficient as stand-alone evidence of reliability (O’Connor & Joffe, 2020). Differences among the reviewers were documented, and the relevant meaning units were reconsidered within the context of the original interviews. Final decisions were reached through discussion and consensus, and the consensus-based coding structure was used when reporting the findings.

The frequencies presented in the qualitative tables represent the number of statements coded within each category. Because a participant could contribute more than one codable statement and the same meaning unit could be assigned more than one code, these frequencies were not interpreted as numbers or proportions of participants. They were used descriptively to show the distribution of the coded statements, rather than to rank the prevalence or importance of the themes. Direct quotations were selected from the final coded dataset to illustrate the scope and diversity of participants’ experiences. The participant quotations presented in the Findings section were translated from Turkish into English by one of the researchers. The second researcher compared each translation with the corresponding original Turkish statement to check whether its meaning, context, and tone had been preserved. Any revisions considered necessary were made through joint discussion and agreement between the two researchers. For culturally specific expressions without a direct English equivalent, the closest contextually appropriate wording was used to preserve the intended meaning rather than reproduce the expression word for word; the final wording was agreed upon by both researchers.

#### 2.5.3. Integration of Quantitative and Qualitative Data

After the quantitative and qualitative data had been analyzed using their respective methods, related findings were considered together within the same research questions. Results concerning the scales’ overall and subscale scores were compared with the themes and categories derived from the interviews in joint displays. During integration, each quantitative–qualitative pairing was first examined to determine whether the two sources represented the same or sufficiently related constructs. Findings that reflected different constructs or time frames were not interpreted as divergent solely because they differed in direction.

Because Questions 1–4 and 6–8 were explicitly informed by the constructs assessed by the quantitative instruments, part of the conceptual correspondence between the two datasets was built into the design of the interview guide. Similar patterns across the two sources were therefore interpreted as conceptual correspondence and contextual explanation rather than as independent corroboration or triangulation. Participants’ retrospective accounts of their reading practices in response to Question 5 and their accounts of roles and implementation processes in response to Questions 9–11 were not treated as direct qualitative counterparts of the quantitative attitude scores.

## 3. Results

This section presents the findings concerning preservice Turkish language teachers’ reading attitudes and attitudes toward book-reading habits following the literature circle program. Consistent with the mixed-methods design, the results were not limited to separate reports of the quantitative and qualitative data. Instead, for each research question, the quantitative results were considered alongside qualitative findings that provided contextual explanations and further insight. This approach offered an integrated account based on both the statistical results obtained from the measurement instruments and participants’ experiences. The findings are presented in three stages. First, the quantitative and qualitative findings concerning reading attitudes are reported and then integrated. Second, the findings concerning attitudes toward book-reading habits are addressed using the same integrative approach. Finally, the quantitative and qualitative findings are considered together to examine where the two data sources addressed related constructs and where they captured different aspects of participants’ experiences.

### 3.1. Findings on Reading Attitudes Following the Literature Circle Program

This section presents the findings concerning preservice Turkish language teachers’ reading attitudes following the literature circle program. First, the pretest and posttest scores obtained from the Reading Attitude Scale for Preservice Teachers were compared. The qualitative findings derived from participants’ accounts of their literature circle experiences are then presented. Finally, the quantitative and qualitative findings are integrated to examine how participants’ experiences of the program corresponded with the observed reading attitude results.

#### 3.1.1. Quantitative Findings on Reading Attitudes

Paired-samples *t*-tests were conducted to examine the pretest–posttest differences in preservice teachers’ reading attitude scores. The results are presented in Table 2.

The overall reading attitude score increased from 138.25 at pretest to 145.81 at posttest. The estimated mean change was 7.56 points, 95% CI [2.09, 13.02], t = 2.81, unadjusted *p* = 0.008. The standardized change was *d*_z_ = 0.47, 95% CI [0.12, 0.81]. However, this comparison did not meet the 0.05 threshold after the Holm adjustment; Holm-adjusted *p* = 0.057. For interest in and enjoyment of reading, the estimated mean change was 3.89 points, 95% CI [0.68, 7.10], t = 2.46, unadjusted *p* = 0.019, *d*_z_ = 0.41, 95% CI [0.07, 0.75]. This comparison also did not remain statistically significant after the Holm adjustment, Holm-adjusted *p* = 0.113. For perceived importance and value of reading, the estimated mean change was 2.97 points, 95% CI [1.12, 4.83], t = 3.25, unadjusted *p* = 0.003, *d*_z_ = 0.54, 95% CI [0.19, 0.89]. This was the only paired-samples *t*-test comparison that met the significance threshold after the Holm adjustment across the eight comparisons, Holm-adjusted *p* = 0.020. For contribution to personal and social development, the estimated mean change was 0.69 points, 95% CI [−2.63, 4.02], t = 0.42, unadjusted *p* = 0.674, Holm-adjusted *p* = 1.000, *d*_z_ = 0.07, 95% CI [−0.26, 0.40]. The confidence intervals for this dimension included values compatible with both a decrease and an increase.

Diagnostic checks of the change scores yielded skewness values ranging from −0.25 to 0.66 and excess kurtosis values ranging from −0.83 to 3.30. The Shapiro–Wilk tests did not indicate a clear departure from normality for contribution to personal and social development (W = 0.966, *p* = 0.334) or overall reading attitude (W = 0.973, *p* = 0.519). Departures from normality were observed, however, for interest in and enjoyment of reading (W = 0.875, *p* < 0.001) and perceived importance and value of reading (W = 0.901, *p* = 0.004). Boxplot screening using the 1.5 interquartile-range criterion flagged four potential outlying change scores for interest in and enjoyment of reading and two for overall reading attitude. Only one change score, in the interest and enjoyment dimension, exceeded the prespecified standardized threshold of |z| > 3.29. The corresponding item responses fell within the permissible range of the scale, and the observation was retained in the analyses. None of the four reading-attitude outcomes contained scores at the theoretical minimum at either measurement point. The proportions of scores at the theoretical maximum ranged from 0% to 8.3%.

The Wilcoxon signed-rank sensitivity analyses produced a pattern that differed in part from the paired-samples *t*-test results after the Holm adjustment. The difference in overall reading attitude remained statistically significant, W = 125.50, unadjusted *p* = 0.006, Holm-adjusted *p* = 0.045. For interest in and enjoyment of reading, the unadjusted result met the conventional 0.05 threshold, but the adjusted *p* value remained slightly above this threshold, W = 112.50, unadjusted *p* = 0.008, Holm-adjusted *p* = 0.055. The difference in perceived importance and value of reading likewise did not meet the significance threshold after the Holm adjustment, W = 111.50, unadjusted *p* = 0.013, Holm-adjusted *p* = 0.076. No statistically significant difference was identified for contribution to personal and social development, W = 233.00, unadjusted *p* = 0.768, Holm-adjusted *p* = 1.000. These findings indicate that the inferences concerning overall reading attitude, interest in and enjoyment of reading, and perceived importance and value of reading were sensitive to both the analytic method and the correction for multiple comparisons.

#### 3.1.2. Qualitative Findings on Reading Attitudes

Participants’ accounts were analyzed to examine how they perceived the relationship between their literature circle experiences and their reading attitudes. Because the interviews were conducted only after the program, comparisons participants made between their earlier reading practices and their experiences during the program were treated as retrospective self-assessments rather than directly observed temporal changes. The analysis generated three overarching themes: cognitive and strategic reading awareness, affective and value-based orientation toward reading, and social interaction and expressive skills. The themes, categories, and codes derived from participants’ accounts are presented in Table 3.

As shown in Table 3, one pattern in participants’ accounts concerned their perception that they approached texts more purposefully and strategically. When retrospectively describing their reading before the program, participants referred mainly to following the plot or attempting to grasp the general meaning. In contrast, they reported paying closer attention during the program to symbols, key concepts, characters’ perspectives, and implicit meanings. Because these comparisons were not supported by qualitative data collected before the program, they represent participants’ retrospective perceptions rather than directly observed change.

Accounts within the in-depth reading category indicated that participants perceived themselves as reading more slowly, carefully, and purposefully. One participant stated, *“I read books before, too, but I had never read with this much engagement or understanding. The greatest benefit for me was learning how to read a book.”* (K_23). Another participant described the perceived change in her reading as follows: *“Before this, I realized that I used to read almost absent-mindedly, without paying attention to words, symbols, or especially meaning. Now I can find meaning even in every word I read.”* (K_3).

These accounts described a perceived shift from reading primarily to finish a book toward identifying different layers of meaning in a text. The statement *“I had never read a book in such detail within such a short time before.”* (E_5) further illustrates this pattern. Taken together, these accounts suggest that participants approached reading more carefully, closely, and analytically during the program.

Another dimension of cognitive and strategic reading awareness was critical and multidimensional thinking. Participants reported that, in their reading during the program, they considered not only the explicit content of the texts but also the characters’ actions, the reasons underlying events, symbolic meanings, and connections between the texts and everyday life. These accounts reflect participants’ perceptions that they approached reading from a broader range of perspectives during the program. One participant explained, *“It taught me reading techniques, different perspectives and meanings, and how to apply what I read to my own life.”* (K_21). This statement illustrates how the participant perceived the process as adding greater intellectual depth to the reading experience.

A similar pattern was evident in the meanings participants attributed to books. One participant stated, *“Honestly, I used to see books as something simple. Now I know that, for me, books are gateways to other worlds—things that help me discover myself and view life from different perspectives.”* (E_8). In this account, reading was understood not merely as a means of acquiring information or completing a text, but as an experience that enabled the participant to consider both the self and life from different perspectives.

The systematic and methodical reading category encompassed participants’ accounts of the guiding role of the role sheets in helping them approach texts with a specific purpose and in a more structured manner. In these accounts, participants reported attending not only to the question “What am I reading?” but also to “How am I reading?” Reading was therefore described in their retrospective evaluations as a more planned, responsibility-based, and meaning-oriented experience.

The second overarching theme, affective and value-based orientation toward reading, encompassed participants’ accounts of their interest, enjoyment, willingness to read, and awareness of the value of reading during the program. Participants associated encountering different books, noticing details in the texts, and discussing the books within their groups with a more engaging reading experience. In their accounts, reading was described not only as an individual obligation but also as an enjoyable and shareable activity.

One participant stated, *“Reading a book each week increased my interest, and I began to enjoy reading more. The way I approached and evaluated a book changed, and I started engaging in these activities more willingly and enthusiastically.”* (K_8). Another explained, *“Noticing details in books that I had previously overlooked sparked my interest in reading.”* (E_3). These accounts suggest that participants’ interest in reading was associated not only with the subject matter of the books but also with the experience of discovering previously unnoticed details in the texts.

Participants’ accounts also reflected a stronger awareness of the value of reading. They reported becoming more conscious of the importance of reading for personal development, empathy, thinking skills, and cultural knowledge. One participant stated, *“It made me realize that reading books is not merely a habit or an obligation, but part of a culture and an important part of our lives.”* (K_1). In this account, the participant interpreted reading as a broader life practice.

This awareness of value was also evident when participants associated reading with empathy and perspective taking. One participant explained, *“Through reading, I learned to view events from different characters’ perspectives, empathize with them, understand how to approach situations, and see them from another angle.”* (K_12). This statement suggests that the participant associated the reading experience not only with approaching texts differently but also with reconsidering how people and events could be understood.

The third overarching theme, social interaction and expressive skills, reflected participants’ experiences of literature circles as a social learning environment. The within-group interaction and collaboration category encompassed experiences of discussing books together, listening to different perspectives, following group norms, and developing a shared reading experience. Participants thus described reading not solely as an individual activity but also as a space for social exchange.

One participant described the process as follows: *“Meeting with my groupmates every week to talk about a book and enjoy ourselves had a positive influence on us. It strengthened our social bonds.”* (K_5). This account shows that the participant associated the literature circle experience not only with engagement in the text but also with social bonds among group members. Participants also described group discussions as opportunities to reconsider what they had read and compare their own interpretations with those of others.

Within the self-expression and communication category, participants described sharing opinions, speaking in public, offering interpretations, and expressing their ideas more comfortably. One participant stated, *“The literature circle helped me recognize the importance of working as a group and supported the development of my communication skills.”* (K_19). Similarly, another reported, *“Working in a group with my friends improved both my communication skills and my social development.”* (E_1). These statements illustrate participants’ perceptions of the literature circle process as supporting communication alongside social interaction.

Another aspect of the social interaction and expressive skills theme was empathy and understanding different perspectives. Participants described group discussions as opportunities not only to present their own interpretations but also to listen to others and consider the texts from different perspectives. In their accounts, they reported extending the meanings they constructed from the books through other participants’ interpretations and considering alternative viewpoints. Taken together, these findings show how participants associated their literature circle experiences with cognitive, affective, and social dimensions of reading attitudes.

#### 3.1.3. Joint Interpretation of Reading Attitude Findings

Before the quantitative and qualitative findings were integrated, the correspondence between the scale dimensions and the qualitative themes was examined. The two sets of findings were not treated as measuring identical aspects of reading attitude. The scale scores represented pretest–posttest comparisons, whereas the interviews captured participants’ retrospective accounts of their experiences during the program. Only qualitative findings that showed a clear conceptual relationship with a scale dimension were included in the joint display. Because the interview questions were partly informed by the scale dimensions, the qualitative findings were used to contextualize rather than independently confirm the quantitative results. The integrated findings are presented in Table 4.

The clearest cross-method correspondence concerned the perceived importance and value of reading. The increase in this dimension remained statistically significant after the Holm adjustment, and participants’ accounts addressed conceptually related issues such as the place and value of reading in their lives. This pattern indicates correspondence between the two sources, although the single-group design does not permit the observed increase to be attributed specifically to the literature-circle program.

For interest in and enjoyment of reading, both the quantitative point estimate and the qualitative accounts were favorable in direction. However, the quantitative comparison did not remain statistically significant after the Holm adjustment. Participants’ accounts of curiosity, enjoyment, and willingness to read may help explain how they experienced the program, but they do not independently establish a change in this dimension.

The contribution to personal and social development dimension showed limited direct correspondence across the two sources. The quantitative estimate was small and imprecise, whereas participants described communication, collaboration, empathy, and self-expression during the group activities. These accounts concerned experiences within the literature-circle setting and were not equivalent to a change in the broader reading-attitude dimension assessed by the scale. The difference between the findings should therefore not be treated as a direct contradiction.

Overall, the qualitative findings elaborated on participants’ experiences of reading and group interaction, while the quantitative findings estimated pretest–posttest differences in the corresponding scale scores. Given the post-program timing of the interviews and the partial alignment of the interview questions with the scale dimensions, the qualitative findings were used primarily to provide context. They were not treated as independent corroboration of the quantitative results.

### 3.2. Findings on Attitudes Toward Book-Reading Habits Following the Literature Circle Program

This section presents the findings concerning preservice Turkish language teachers’ attitudes toward book-reading habits following the literature circle program. First, the pretest and posttest scores obtained from the Preservice Teachers’ Attitudes toward Book-Reading Habits Scale were compared. The qualitative findings derived from participants’ accounts are then presented. Finally, the quantitative and qualitative findings are integrated to examine how participants’ experiences of the program corresponded with the results for attitudes toward book-reading habits.

#### 3.2.1. Quantitative Findings on Attitudes Toward Book-Reading Habits

Paired-samples *t*-tests were conducted to examine the pretest–posttest differences in preservice teachers’ attitudes toward book-reading habits. The results are presented in Table 5.

The overall attitude score increased from 118.03 at pretest to 120.67 at posttest. The estimated mean change was 2.64 points, 95% CI [−5.39, 10.67], t = 0.67, unadjusted *p* = 0.509, Holm-adjusted *p* = 1.000. The standardized change was *d*_z_ = 0.11, 95% CI [−0.22, 0.44]. None of the three dimension-level comparisons met the statistical significance threshold. For meeting learning needs and enjoyment, the estimated mean change was 1.50 points, 95% CI [−3.61, 6.61], t = 0.60, unadjusted *p* = 0.555, Holm-adjusted *p* = 1.000, dz = 0.10, 95% CI [−0.23, 0.43]. For meaning and indispensability of book-reading habits, the estimated mean change was 0.39 points, 95% CI [−1.65, 2.43], t = 0.39, unadjusted *p* = 0.701, Holm-adjusted *p* = 1.000, *d*_z_ = 0.06, 95% CI [−0.26, 0.39]. For development of book-reading habits, the estimated mean change was 0.75 points, 95% CI [−0.71, 2.21], t = 1.05, unadjusted *p* = 0.303, Holm-adjusted *p* = 1.000, *d*_z_ = 0.17, 95% CI [−0.16, 0.50]. The effect-size estimates were small, and all confidence intervals included zero. Accordingly, the estimates were interpreted as imprecise and compatible with values in either direction rather than as conclusive evidence that no change occurred.

For the change scores concerning attitudes toward book-reading habits, skewness values ranged from −0.12 to 0.24, and excess kurtosis values ranged from −0.85 to −0.03. The Shapiro–Wilk tests did not indicate a clear departure from normality for meeting learning needs and enjoyment (W = 0.955, *p* = 0.152), meaning and indispensability of book-reading habits (W = 0.954, *p* = 0.138), development of book-reading habits (W = 0.983, *p* = 0.845), or the overall score (W = 0.965, *p* = 0.308). Boxplot screening flagged three potential outlying change scores for meaning and indispensability of book-reading habits, but none exceeded the prespecified standardized threshold of |z| > 3.29. These observations were retained in the analyses. No scores were observed at the theoretical minimum at either measurement point, whereas the proportions of scores at the theoretical maximum ranged from 0% to 5.6%.

The Wilcoxon signed-rank sensitivity analyses did not indicate statistically significant differences in the overall score (W = 236.50, unadjusted *p* = 0.607), meeting learning needs and enjoyment (W = 210.00, unadjusted *p* = 0.643), meaning and indispensability of book-reading habits (W = 189.50, unadjusted *p* = 0.758), or development of book-reading habits (W = 169.00, unadjusted *p* = 0.293). After the Holm adjustment was applied across all eight comparisons, the adjusted *p* value was 1.000 for each of these four outcomes. The sensitivity analyses were therefore broadly consistent with the paired-samples *t*-test results and did not provide clear evidence of a pretest–posttest difference in the overall score or any of the three dimensions of attitudes toward book-reading habits.

#### 3.2.2. Qualitative Findings on Participants’ Reported Reading Practices and Experiences

Participants’ accounts focused on the weekly reading routines they reported following during the program, their willingness to read, more purposeful engagement with texts, and sharing what they had read within their groups. These accounts were treated not as evidence of directly observed reading behavior or the development of a lasting reading habit, but as participants’ retrospective evaluations provided after the program. The findings were organized into three overarching themes: reading routines and willingness to read during the program, reported strategic and purposeful reading practices, and social learning and sharing. The themes, categories, and codes derived from participants’ accounts are presented in Table 6.

The first theme presented in Table 6 concerned the weekly reading routines that participants reported following during the program and their willingness to read. Participants associated the weekly reading schedule and group responsibilities with allocating time to reading and proceeding in a more planned manner throughout the program. They described the weekly structure as helping them sustain reading within a defined schedule and framework of responsibility rather than treating it as an activity that could easily be postponed. These statements describe the reading routine participants perceived during the program; they do not constitute direct measurement of behavior outside or after the program.

One participant stated, “*Reading one book a week helped us establish a routine and develop a habit. We made our book reading more disciplined*.” (E_1). Another similarly explained, “*Reading a book regularly every week and discussing it both improved my reading habits and, as I discussed what I had read with my friends, improved my social and communication skills*.” (K_26). References to developing or improving a habit in these quotations represent the participants’ own interpretations. Because reading frequency and time spent reading were not independently monitored and no follow-up assessment was conducted, these accounts were not treated as evidence that a lasting reading habit had developed. Instead, they illustrate how participants associated the weekly program structure, group responsibilities, and opportunities for sharing with a more regular reading experience.

Another topic considered alongside reading routines during the program was willingness to read and intrinsic interest. When describing their experiences, some participants reported greater willingness to read books and greater enjoyment of reading. In their accounts, the weekly reading schedule and favorable evaluations of reading appeared together. However, because the interviews were conducted during the week after the program, the reported willingness does not show that reading behavior continued over a longer period. One participant described this perceived change emphatically: “*It improved it so, so, so much. I did not really have much of a reading habit, but now I want to read books all the time*.” (K_19). Another highlighted both learning and enjoyment: “*I realized that I need to read in order to learn and that reading can also be enjoyable*.” (K_3). These statements reflect participants’ self-assessments of their willingness to read and the meaning they attributed to reading at the time of the interview; they are not direct indicators of lasting behavioral change.

The theme of reported strategic and purposeful reading practices encompassed participants’ self-assessments of how they worked with texts. Participants described not only how often they read but also experiences of examining books more carefully, taking notes, interpreting symbols, questioning what they read, and conducting additional research when needed. These accounts indicate how participants believed they approached reading during the program; they do not imply that changes in reading behavior were independently measured. The methodological reading awareness category included participants’ accounts of how the role sheets structured the reading process. They reported that assuming a different role each week and reading with that role in mind helped them approach each book with a clearer purpose. One participant stated, “*One of the greatest things it gave us was undoubtedly learning how a book should be read. I used to read straight through without much thought, but now I have learned how to read a book properly*.” (K_14).

Within the comprehension and critical reading category, participants described adopting a more questioning approach to reading. One participant stated, *“Before this activity, I was someone who simply read and moved on, but now I can say that I have become someone who researches and questions while reading.”* (K_11). Another explained, *“I used to get bored when reading and read books only superficially. Through the literature circle, I realized that I had begun to question, critique, take notes, learn new things, and enjoy reading.”* (K_4). These accounts suggest that participants understood their literature circle experiences in terms of reading quality as well as reading frequency. Reading was described as a more active experience involving questioning, analysis, note-taking, research, and meaning-making rather than merely finishing a book.

The pedagogical and methodological awareness category encompassed participants’ accounts of relating the literature-circle process to their future professional roles. Participants described how a book could be read and discussed and how the reading process could be structured. These accounts reflected both the consideration of their own reading practices and their experience of an instructional approach that they might use with their future students.

The third overarching theme, social learning and sharing, encompassed participants’ experiences of discussing books within a group, listening to different ideas, and sharing what they had read. Participants associated these interactions with a more engaging reading process that was easier to sustain. In their accounts, book reading was framed not only as an individual behavior but also as an experience supported by social interaction. Within the peer discussion and sharing category, participants described opportunities to consider what they had read together with others. One participant stated, *“Thanks to the literature circle, we talked and debated a great deal about books, and I really enjoyed it.”* (K_7). Another explained, *“Because the literature circle was conducted as a group, talking about the book was both informative and enjoyable.”* (K_25). These accounts highlight the affective value that participants attributed to sharing their reading experiences and the learning and enjoyment associated with group discussions.

Solidarity, communication, and self-expression also emerged within the social learning and sharing theme. Participants reported that assuming responsibility within the group, explaining their ideas, and listening to different perspectives made the reading process more meaningful. Social interaction therefore appeared in their accounts as a source of motivation associated with reading. Discussions of the books allowed participants not only to construct their own interpretations but also to extend those interpretations through the perspectives of others.

#### 3.2.3. Joint Interpretation of Findings on Attitudes Toward Book-Reading Habits

Before the findings were integrated, the conceptual correspondence between the dimensions of the attitude scale and the qualitative themes was examined. The two sources were not treated as equivalent measures. The quantitative findings represented pretest–posttest differences in attitudes toward book-reading habits, whereas the interviews captured participants’ retrospective accounts of their experiences during the program. Qualitative findings were included in the joint display only when their content was conceptually related to a scale dimension. Because the interviews were conducted only after the program and some interview questions were informed by the scale dimensions, the qualitative accounts were used to contextualize rather than independently confirm the quantitative findings. The integrated findings are presented in Table 7.

The quantitative estimates for the total score and all three dimensions were small, and their confidence intervals included zero. These results did not provide clear evidence of a pretest–posttest change in attitudes toward book-reading habits in the present sample. At the same time, the width of the confidence intervals does not support a firm conclusion that no change occurred. Participants’ qualitative accounts described favorable experiences during the program. They described reading according to a weekly schedule, allocating time to books, engaging with texts more deliberately, and sharing their interpretations with peers. These accounts may be relevant to processes associated with the development of reading habits, but they do not show whether the reported routines continued after the program or developed into stable reading behavior.

The relationship between the two sources was therefore interpreted as complementary but only partially aligned. The qualitative findings described participants’ perceived experiences, whereas the quantitative findings estimated changes in scale scores. In particular, reports of reading regularly and following a weekly schedule referred partly to behavior within the program, while the scale assessed attitudes toward book-reading habits. This difference in construct and timing limits claims of direct convergence or divergence. Participants associated their experiences with several elements of the program, including the weekly reading schedule, selected books, role sheets, rotating responsibilities, group discussions, and peer interaction. Because these components were implemented together, the present findings do not show which component was related to a particular experience or score pattern. The integrated findings should therefore be interpreted as an account of the program as a whole rather than evidence concerning the effect of an individual component.

## 4. Discussion

This study examined pretest–posttest differences in preservice Turkish language teachers’ reading attitudes and attitudes toward book-reading habits and considered the quantitative findings alongside participants’ accounts of their literature-circle experiences. Positive change estimates were observed for overall reading attitude, interest in and enjoyment of reading, and perceived importance and value of reading. However, the outcome that met the conventional threshold for significance after the Holm adjustment differed between the paired-samples *t* test and the Wilcoxon sensitivity analysis, indicating that the inferential conclusion was sensitive to the analytic method. Change estimates were small and imprecise for contribution to personal and social development and for all outcomes related to attitudes toward book-reading habits. Participants described favorable experiences involving interest, awareness of the value of reading, more deliberate engagement with texts, regular reading, and peer interaction. Because these accounts were collected only after the program and some interview questions drew on the scale dimensions, they were interpreted as contextual evidence of participants’ experiences rather than as independent confirmation of quantitative change. The single-group design also precludes attributing the observed changes specifically to the literature-circle program.

### 4.1. Discussion of the Findings on Reading Attitudes

The quantitative findings did not indicate a uniform pattern across the dimensions of reading attitude. The clearest result concerned perceived importance and value of reading. The estimated mean increase in this dimension was 2.97 points, with a standardized change of *d*_z_ = 0.54, and the comparison remained statistically significant after the Holm adjustment. The corresponding confidence interval did not include zero, although it indicated uncertainty regarding the precise magnitude of the change. The estimates for overall reading attitude and interest in and enjoyment of reading were also positive, but these comparisons did not meet the 0.05 threshold after adjustment for multiple testing. This distinction limits support for a broad improvement in reading attitude and suggests that the quantitative evidence was more specific to participants’ perceptions of the importance and value of reading.

The positive direction of the overall reading-attitude estimate is broadly consistent with studies reporting favorable outcomes following literature-circle programs (Doğan et al., 2020; Nurisma, 2025). Su and Jia (2026) and Teng et al. (2024) also identified aspects of literature circles that participants evaluated favorably, although these studies focused on attitudes toward the instructional approach rather than general reading attitudes. Other studies conducted with different participant groups and educational contexts did not identify statistically significant differences in reading-attitude scores (Çağ, 2024; Demir & Pilav, 2023; Doğan et al., 2021). The present result falls between these patterns because the overall reading-attitude estimate was positive but sensitive to adjustment for multiple comparisons. Differences among studies may be associated with participant characteristics, program duration, selected texts, measurement instruments, or implementation conditions. The present design did not compare these explanations directly.

The quantitative and qualitative findings showed their closest conceptual correspondence in relation to the perceived importance and value of reading. Participants described reconsidering the place of reading in their lives and becoming more aware of its cultural, intellectual, and personal value. These accounts were consistent in content with the quantitative increase in the corresponding scale dimension. Participants also reported greater willingness to read, curiosity about different books, and enjoyment of reading and group discussions. Previous studies have considered literature circles in relation to reading culture, motivation, engagement, and enjoyment (Allan et al., 2005; Baki, 2023; Khonamri et al., 2024; Schoonmaker, 2014). Linde and Daniela (2025) similarly reported favorable teacher perceptions of literature-circle activities used alongside graded readers. These studies provide a context for participants’ accounts, but their outcomes and implementation settings were not identical to those of the present study. Moreover, the interest and enjoyment comparison did not remain statistically significant after the Holm adjustment. Because the interviews were retrospective and some questions were informed by the scale dimensions, the qualitative accounts were interpreted as descriptions of participants’ experiences rather than independent confirmation of quantitative change.

A different relationship was observed for contribution to personal and social development. The quantitative estimate was close to zero, and its confidence interval included values in both directions. Participants nevertheless described communication, collaboration, self-expression, empathy, and listening to different perspectives during group activities. Research on literature circles has examined similar experiences in relation to participation, democratic discussion, collaborative learning, social skills, group interaction, self-expression, and encountering different viewpoints (Aytan, 2018; Brown, 2015; Çermik et al., 2019; Çetinkaya & Topçam, 2019; Doğan et al., 2019; Kang et al., 2023; Kaya Tosun, 2018; Venegas, 2019; Widodo, 2016). These studies help contextualize the social experiences described by participants. However, such program-specific experiences are not identical to the broader attitude construct assessed by the contribution to personal and social development dimension. The two sources were therefore interpreted as partially complementary rather than directly convergent or contradictory.

Cognitive and strategic reading awareness was identified in the qualitative findings but was not a separate dimension of the Reading Attitude Scale. Participants described examining texts in greater detail, attending to symbols and key concepts, considering alternative interpretations, and using role sheets to approach reading more systematically. Related experiences have been examined in studies addressing academic reading, critical and multidimensional thinking, creative and detailed reading, engagement with texts, reading comprehension, and reading strategies within literature-circle contexts (Ament et al., 2025; Baki, 2022, 2025; Çermik et al., 2019; Cloonan et al., 2020; Doğan et al., 2019; Karatay & Soysal, 2021; Ma, 2025; Mrak, 2021). These accounts add detail concerning how participants experienced the reading process, but they should not be treated as evidence that strategic reading caused the observed attitude-score pattern.

Overall, the findings do not support a uniform improvement across all dimensions of reading attitude. The most consistent quantitative evidence concerned the perceived importance and value of reading. The positive estimates for overall reading attitude and interest and enjoyment remain potentially relevant, but their interpretation is less certain after correction for multiple comparisons. The qualitative findings add contextual detail concerning participants’ experiences of interest, value, strategic reading, and social interaction. The selected books, weekly reading schedule, rotating roles, role sheets, and group discussions were implemented together; therefore, the study cannot identify which component, if any, was associated with a particular experience or score pattern. In addition, the single-group design does not support attributing the observed quantitative pattern specifically to the literature-circle program.

### 4.2. Discussion of the Findings on Attitudes Toward Book-Reading Habits

The estimated mean changes in the overall score and the three dimensions of attitudes toward book-reading habits were small, and all of the confidence intervals included zero. The paired-samples *t* tests and the Wilcoxon sensitivity analyses indicated the same general pattern after the Holm adjustment. Thus, the quantitative results did not provide clear evidence of a pretest–posttest difference in scores for attitudes toward book-reading habits over the seven-week period in this sample. Nevertheless, because the confidence intervals were compatible with both decreases and increases, the findings should not be interpreted as conclusive evidence that no change occurred.

This quantitative pattern is similar to the findings of Çağ (2024), who identified no statistically significant difference in book-reading habits among fifth-grade students. By contrast, Serin and Turan (2022) reported a statistically significant difference in preservice teachers’ attitudes toward book-reading habits in a nine-week study that included a control group. Variation across the studies may be related to participant characteristics, program duration, selected books, course context, or implementation conditions. However, because these possibilities were not directly compared in the present study, the different findings cannot be attributed to any particular factor.

The interviews offered a more favorable picture of participants’ experiences. Participants reported following a weekly reading schedule, allocating time to reading, approaching books more purposefully, and benefiting from sharing what they had read with their peers. Comparable experiences have been reported in studies associating literature circles with regular reading, reading culture, and reading habits (Avcı et al., 2010; Baki, 2023; Doğan et al., 2018, 2021). However, the interviews in the present study were conducted only after the program, and neither reading frequency nor time devoted to reading was independently measured. These accounts should therefore not be interpreted as behavioral evidence that a lasting book-reading habit developed or that participants’ attitudes toward such habits changed.

Considered together, the two strands indicate the need to distinguish structured reading practices during the program from broader attitudinal orientations toward book-reading habits. The weekly schedule, role responsibilities, and accountability to peers may have supported participants in continuing to read throughout the seven-week program. In contrast, the scale did not measure whether participants completed the books during the program; it assessed their broader evaluations of the regularity, meaning, indispensability, and development of book reading (Sarar Kuzu & Doğan, 2015; Susar Kırmızı, 2012). This interpretation does not represent a directly tested mechanism, but it provides a conceptual explanation for why favorable program experiences may not have been reflected to the same extent in attitude scores over a relatively short period. The qualitative accounts do not override the quantitative findings, just as the uncertainty in the quantitative estimates does not imply that the program was without value to participants. The two strands provide information about related but non-equivalent phenomena.

### 4.3. Integrated Discussion of the Quantitative and Qualitative Findings

When considered together, the quantitative and qualitative findings suggest that reading attitude and attitudes toward book-reading habits should be treated as related but distinct constructs. Positive change estimates were observed for overall reading attitude, interest in and enjoyment of reading, and perceived importance and value of reading, whereas the change in contribution to personal and social development was very small. No clear pretest–posttest difference emerged for the overall score or any of the three dimensions of attitudes toward book-reading habits. The quantitative findings therefore do not support a common pattern of improvement across both outcome domains.

The reading-attitude findings also need to be viewed in light of the difference between the two analytic approaches. After the Holm adjustment, perceived importance and value of reading met the significance threshold in the paired-samples *t* test, whereas overall reading attitude met this threshold in the Wilcoxon sensitivity analysis. Interest in and enjoyment of reading changed in a favorable direction, but its adjusted *p* value remained above 0.05 in both analyses. The overall pattern is therefore better interpreted by considering the direction and magnitude of the estimates, their confidence intervals, and their sensitivity to the analytic method, rather than relying only on whether an individual result crossed the significance threshold.

The quantitative and qualitative strands captured different aspects of participants’ experiences. The quantitative analyses estimated pretest–posttest changes in scale scores, while the interviews documented participants’ perceptions after the program. Participants described reconsidering the place and value of reading in their lives, becoming more interested in different books, and finding reading more enjoyable. These accounts were consistent in content with the favorable direction of the reading-attitude estimates. However, the interviews were conducted only after the program, and some questions were informed by the scale dimensions. The qualitative accounts therefore provide context for understanding the quantitative pattern but do not independently confirm that a change occurred.

The relationship between the two strands was less direct for contribution to personal and social development. Participants’ accounts of communication, collaboration, empathy, self-expression, and listening to different viewpoints described social experiences during group activities. The corresponding scale dimension assessed broader attitudes toward the personal and social contribution of reading. Similarly, accounts of following a weekly schedule, setting aside time for books, and fulfilling group responsibilities referred to activities undertaken during the program, whereas the book-reading-habits scale assessed attitudes toward such habits. These findings concern related but nonidentical constructs and are therefore best understood as partially complementary rather than directly convergent or contradictory.

Participants’ accounts of examining texts closely, attending to symbols, developing alternative interpretations, and reading more systematically with the support of role sheets also reflected cognitive and strategic experiences that were not assessed as a distinct dimension of either scale. These findings add detail to the ways in which participants experienced reading during the program, but they do not establish a lasting change in reading attitudes or book-reading habits. Without a follow-up assessment, it is also unclear whether the reading routines described during the program continued after it ended.

Overall, the integrated findings offer a differentiated picture rather than evidence of a single, broad program effect. Quantitative changes in several reading-attitude outcomes were favorable but sensitive to the analytic method, while the estimates for attitudes toward book-reading habits remained small and imprecise. The qualitative findings added detail about participants’ experiences of interest, value, strategic reading, group interaction, and regular reading. Because book selection, the weekly reading schedule, role sheets, rotating responsibilities, and group discussions were implemented together, no particular experience or score pattern can be linked to a single program component. Moreover, without a comparison group, the observed changes cannot be attributed specifically to the literature-circle program.

## 5. Limitations

The findings of this study should be interpreted in light of several limitations. The quantitative phase employed a single-group pretest–posttest design without a comparison group or random assignment. Accordingly, the observed score patterns cannot be attributed solely to the literature-circle program. Repeated measurement, other course-related activities, participants’ expectations and experiences during the study period, and changes over time remain plausible alternative explanations. The findings should therefore be interpreted as within-group pretest–posttest differences rather than as evidence of a causal program effect.

The quantitative analyses were based on 36 participants from a single teacher education program. Because the available course cohort determined the sample size, no a priori sample-size calculation was conducted. A sensitivity analysis indicated that, under the assumptions of a two-sided paired-samples *t*-test with α = 0.05 and independent paired observations, a sample of 36 provided 80% power to detect an effect of approximately *d*_z_ = 0.48 or larger. This calculation describes the sensitivity of the design rather than the power associated with the observed effects (Lakens, 2022). Smaller changes may therefore have been difficult to distinguish from sampling variation, and the width of several confidence intervals reflects the limited precision of the estimates.

Participants took part in six reading circles, each consisting of six members and formed according to participants’ preferences. However, the analytic dataset did not retain a group identifier indicating the reading circle to which each participant belonged. Consequently, potential within-group dependence among participants in the same reading circle could not be modeled in the quantitative analyses. Moreover, the availability of only six groups limits the reliable estimation of group-level effects. The findings should therefore be interpreted in light of this uncertainty regarding the independence of individual observations.

The program lasted seven weeks, and no follow-up assessment was conducted. This period may not have been sufficient to determine whether any changes in attitudes or reading routines persisted after the program. The quantitative instruments assessed attitudes rather than directly observed reading behavior. Reading frequency, time spent reading outside the course, independently selected books, book completion, library use, and reading behavior after the program were not monitored. The absence of data on participants’ usual reading frequency before the program also limits the extent to which the findings can be interpreted in relation to their baseline reading behavior. Participants’ reports of reading more regularly or becoming more willing to read should therefore be understood as self-reported experiences rather than evidence of sustained behavioral change.

An additional limitation concerns the fact that the qualitative interviews were conducted by the researcher who taught the participants’ course. Although participants were explicitly informed that participation was voluntary and that their decision whether or not to participate would not affect their academic performance or course grades, the power imbalance inherent in the instructor–student relationship may have led some participants to emphasize positive experiences or limit the expression of critical views. The qualitative findings should therefore be interpreted in light of the researcher’s dual role.

Both the quantitative instruments and the interviews relied on self-report. Responses may have been influenced by recall bias, social desirability, participants’ awareness of the study purpose, or perceived researcher expectations. The interview guide included questions that allowed participants to report an absence of change, articulate different positions concerning reading, and evaluate both positive and negative aspects of the program. Nevertheless, several questions were framed in terms of a possible effect or change. This wording may have increased the salience of change-related experiences in participants’ accounts. Because the interviews were conducted only after the program, participants’ comparisons between their experiences before and during the program constituted retrospective self-assessments rather than directly observed changes. In particular, the reading frequency, time devoted to reading, and number of books completed addressed in Question 5 were not independently measured or verified.

Questions 1–4 and 6–8 were informed by the overall constructs and dimensions assessed by the quantitative instruments. Consequently, some of the conceptual correspondence between the qualitative and quantitative findings reflected the planned alignment of the interview guide with the scales. Although data-driven coding was permitted, experiences outside the domains explicitly prompted by the interview questions may have been less salient. The qualitative findings were therefore interpreted as contextual accounts of participants’ post-program perceptions and experiences rather than as independent confirmation of the quantitative results. Frequencies presented in the qualitative tables represent coded statements rather than numbers or proportions of participants and should not be interpreted as estimates of prevalence.

The literature-circle program combined several elements, including book selection, weekly reading requirements, role sheets, rotating responsibilities, group discussions, peer interaction, and the preparation of final products. Because these elements were implemented together, the study cannot identify the contribution of any individual component. The selected books were not standardized or formally matched in terms of genre, length, linguistic or conceptual difficulty, or intended readership. Differences in page length and thematic content may have shaped participants’ interest, reading experiences, and engagement in group discussions differently. The study therefore does not allow text-specific influences to be separated from those associated with the other components of the literature-circle program.

Weekly role sheets, observer forms, short video excerpts, and product submissions enabled the implementation process to be monitored throughout the program. However, these records were not collected by an independent evaluator using a standardized implementation-fidelity instrument and were not converted into a quantitative fidelity score. In addition, the video recordings covered only approximately 10–15 min of each meeting rather than the sessions in their entirety. The available records therefore indicate that the planned meetings and core implementation components were completed, but they should not be interpreted as independent evidence that an equivalent level of implementation fidelity was achieved across all sessions and groups.

The interpretation of the pretest–posttest comparisons also assumes that the instruments measured the same constructs in the same way at both measurement points. Longitudinal measurement invariance was not examined, and the sample size was insufficient for a stable invariance analysis of the multidimensional measurement models. The pretest–posttest comparisons should therefore be interpreted with this untested assumption in mind. Although internal-consistency coefficients were reported for both measurement points, these estimates do not by themselves establish longitudinal comparability.

The participants were preservice Turkish language teachers from a single university, and 35 of the 36 participants took part in the qualitative phase. Their experiences may differ from those of preservice teachers in other subject areas, institutions, regions, or educational systems. The transferability of the findings should therefore be considered in light of the specific participants and institutional context.

## 6. Conclusions and Recommendations

This study considered within-group pretest–posttest differences in preservice Turkish language teachers’ reading attitudes and attitudes toward book-reading habits alongside participants’ accounts of their literature-circle experiences. Favorable change estimates were observed for overall reading attitude, interest in and enjoyment of reading, and perceived importance and value of reading. After the Holm adjustment, however, the outcome that met the significance threshold differed between the paired-samples *t* test and the Wilcoxon sensitivity analysis. This method-dependent result limits the extent to which the favorable pattern can be interpreted as clear evidence of change in any single dimension. The change in contribution to personal and social development was very small, and neither analytic approach provided clear evidence of a pretest–posttest difference in the overall score or any dimension of attitudes toward book-reading habits. The quantitative findings therefore do not support a uniform pattern of improvement across all measured outcomes.

Participants described favorable experiences involving reflection on the value and place of reading in their lives, interest in different books, more deliberate engagement with texts, adherence to a weekly reading schedule, and the sharing of ideas with peers. These accounts help explain how participants experienced the program, but they do not independently confirm the quantitative changes. Reports of reading according to a weekly schedule and fulfilling group responsibilities largely concerned activities undertaken during the seven-week program. In the absence of follow-up measures and direct indicators of reading behavior, it remains unclear whether these experiences developed into lasting reading habits.

The findings also need to be considered within the limits of the research design. Without a comparison group, the observed score differences cannot be attributed specifically to the literature-circle program. Group identifiers for the six participant-selected reading circles were not retained in the analytic dataset, preventing possible dependence among members of the same group from being modeled. In addition, book selection, the weekly reading schedule, role sheets, rotating responsibilities, and group discussions were implemented together, so no particular finding can be linked separately to any one component. The results are therefore best viewed as an exploratory account of participants’ score patterns and reported experiences.

Within teacher education, literature circles may offer a structured setting in which preservice teachers encounter different books, examine texts through assigned roles, and discuss their interpretations with peers. The present findings, however, do not support an expectation that such activities will produce predetermined gains across all dimensions of reading attitude or attitudes toward book-reading habits. When literature circles are incorporated into teacher education, the intended learning outcomes should be clearly defined, and actual reading behavior should be considered alongside attitudinal outcomes.

Future research would benefit from the use of comparison groups and, where feasible, random assignment to study conditions. Larger samples drawn from multiple institutions and a sufficient number of literature circles would allow possible within-group dependence to be examined more adequately. Retaining group identifiers would also make multilevel or cluster-aware analyses possible. Specifying primary outcomes and the analysis plan before examining the data could reduce uncertainty arising from multiple comparisons. Attitude measures could be complemented by behavioral indicators such as weekly reading time, reading frequency outside class, the number of books completed, voluntary book selection, and library use. Follow-up assessments would help determine whether reported experiences persist beyond the program. Collecting qualitative data both before and after implementation, drawing on observations or reading records, and assessing implementation fidelity could provide a fuller account of participants’ experiences. Examining book selection, role-based preparation, and peer discussion separately or comparatively may also clarify the processes associated with different reading outcomes.

## Figures and Tables

**Figure 1:**
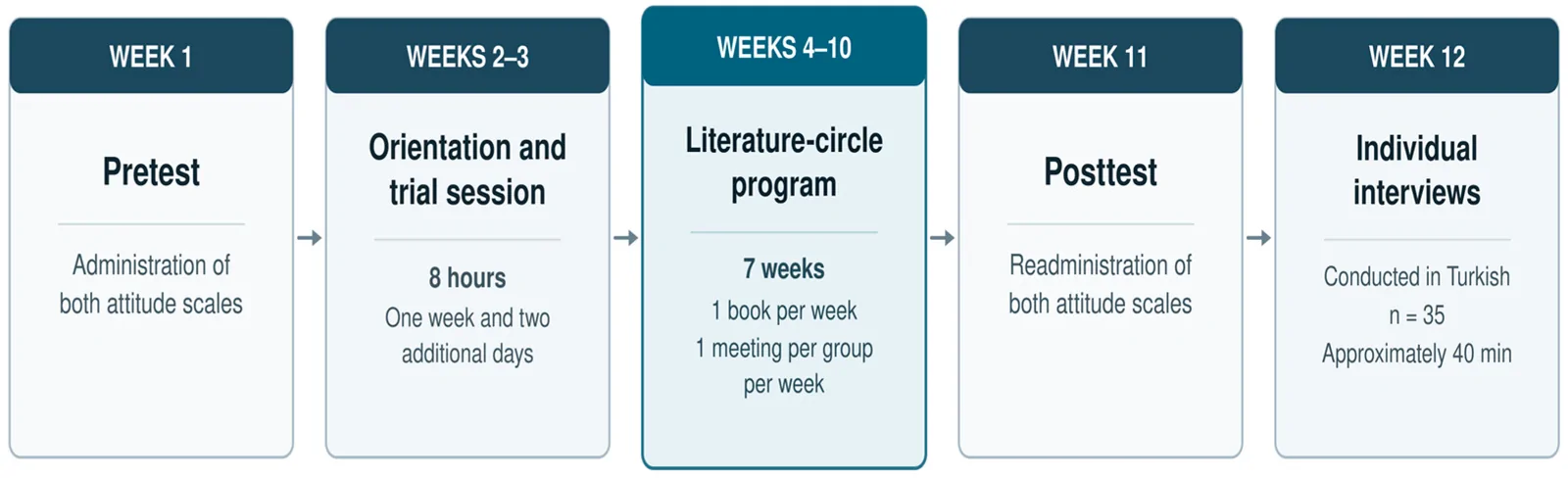
Chronological overview of the 12-week research process.

**Table 1:** Descriptive characteristics of the books read during the program.

Book and Author	Genre and Primary Focus	Length (Pages)	Intended Readership	Descriptive Reading Demands
*Hayalperest Çocuk*—Görkem Kantar Arsoy	Illustrated children’s story; imagination and individual differences	80	Readers aged 9–11	Short, visually supported, and relatively accessible narrative
*Piri Reis ve Göbeklitepe’deki Sır*—Mavisel Yener	Historical adventure novel; exploration, history, and archaeology	144	Children and young readers	Moderate conceptual demands arising from its historical content
*Elleriyle Gören Çocuk*—Tomasz Malkowski	Children’s story; visual impairment, independence, and prejudice	112	Children and early adolescents	Episodic and relatively accessible narrative
*Havanın Rengini Gören Çocuk*—Abdo Wazen	Youth novel; visual impairment, education, and hope	112	Young and adolescent readers	Medium-length narrative with pronounced emotional and reflective content
*Şişko Patates*—Seza Kutlar Aksoy	Fantasy children’s novel; physical differences, labeling, and peer relationships	96	School-age children	Short-to-medium-length and relatively accessible narrative
*Filozof Meşe*—Anooshirvan Miandji	Philosophical and allegorical children’s novel; responsibility, truth, and social life	64	Readers aged approximately 8 years and older	The shortest work, but one that includes abstract and inquiry-oriented content
*Albie Bright’in Sayısız Dünyası*—Christopher Edge	Children’s science-fiction novel; grief, quantum physics, and parallel worlds	180	Middle-grade and young readers	The longest work, with relatively greater conceptual demands arising from its scientific content

*Note.* Page counts are approximate and refer to the Turkish editions; minor variation may occur across printings. Reading difficulty was not assessed using a standardized readability measure. The descriptions in the final column are therefore based only on the length, narrative form, and conceptual content of each work. Intended readership reflects the works’ general publisher and catalog positioning rather than the study sample.

**Table 2:** Pretest–posttest comparisons of reading attitude scores.

Dimension	Pretest M (SD)	Posttest M (SD)	Mean Change [95% CI]	t	*p*	Holm-Adjusted *p*	*d*_z_ [95% CI]
Contribution to personal and social development	83.61 (6.17)	84.31 (8.58)	0.69 [−2.63, 4.02]	0.42	0.674	1.000	0.07 [−0.26, 0.40]
Interest in and enjoyment of reading	39.25 (7.58)	43.14 (7.58)	3.89 [0.68, 7.10]	2.46	0.019	0.113	0.41 [0.07, 0.75]
Perceived importance and value of reading	15.39 (4.86)	18.36 (3.63)	2.97 [1.12, 4.83]	3.25	0.003	0.020	0.54 [0.19, 0.89]
Overall reading attitude	138.25 (14.37)	145.81 (13.23)	7.56 [2.09, 13.02]	2.81	0.008	0.057	0.47 [0.12, 0.81]

*Note.* (*N* = 36) for all comparisons. M = mean; SD = standard deviation; CI = confidence interval. Mean change was calculated as posttest minus pretest. The Holm adjustment was applied across the eight comparisons conducted for the two scales. The reported confidence intervals are pointwise intervals and were not adjusted for multiple comparisons. Consequently, an unadjusted confidence interval may exclude zero even when the corresponding Holm-adjusted *p* value is greater than 0.05.

**Table 3:** Themes, categories, and codes derived from participants’ post-program accounts of reading attitudes.

Theme	Category	Codes	Coded Statements (f)
Cognitive and strategic reading awareness	In-depth reading	Examining the text in detail; reading in depth; recognizing multiple layers of meaning; reading for comprehension	23
	Critical and multidimensional thinking	Analyzing symbols; identifying key concepts; developing different perspectives; interpreting	20
	Systematic and methodical reading	Reading with role sheets; learning how to read a book; experiencing a new approach to reading; reading regularly	7
Affective and value-based orientation toward reading	Interest, enjoyment, and motivation	Increased desire to read; curiosity; enjoyment of reading; devoting more time to reading	8
	Awareness of value	Understanding the place of reading in life; recognizing its cultural value; appreciating the value of knowledge; regarding reading as important	22
Social interaction and expressive skills	Within-group interaction and collaboration	Reading as a group; group discussion; collaborative work; learning group rules; solidarity; forming social bonds	36
	Self-expression and communication	Sharing opinions; speaking in public; giving presentations; asking questions; expressing and interpreting ideas; developing communication skills	17
	Empathy and understanding different perspectives	Empathizing; listening to different perspectives; recognizing others’ viewpoints; broadening one’s perspective	8

*Note.* Frequencies represent the number of statements coded within each category. Because a participant’s response could be coded under more than one code, the frequencies should not be interpreted as numbers of participants. These frequencies should not be used to rank the categories by their prevalence among participants or their relative importance. Comparisons between participants’ reading before and during the program reflect retrospective self-assessments provided after the program and should not be interpreted as directly observed changes.

**Table 4:** Integration of quantitative and qualitative findings on reading attitudes.

Reading Attitude Dimension	Quantitative Finding	Qualitative Finding with the Closest Conceptual Correspondence	Integrated Interpretation
Interest in and enjoyment of reading	The estimated mean increase was 3.89 points, 95% CI [0.68, 7.10], *d*_z_ = 0.41. The unadjusted *p* value was 0.019, but the comparison did not remain statistically significant after the Holm adjustment; adjusted *p* = 0.113.	Participants described greater willingness to read, curiosity about different books, enjoyment of reading, and increased interest associated with discussing books with peers.	The two sources showed a similar directional pattern in relation to interest and enjoyment. However, because the quantitative comparison did not remain significant after the Holm adjustment and the interviews were retrospective, the qualitative accounts were interpreted as contextual information rather than confirmation of a pretest–posttest change.
Perceived importance and value of reading	The estimated mean increase was 2.97 points, 95% CI [1.12, 4.83], *d*_z_ = 0.54. The difference remained statistically significant after the Holm adjustment, adjusted *p* = 0.020.	Participants reported greater awareness of the cultural, intellectual, and personal value of reading and reconsidered the place of reading in their lives.	This dimension showed the clearest correspondence between the two sources. The quantitative increase was accompanied by qualitative accounts concerning the perceived value of reading. Nevertheless, the post-program accounts were treated as descriptions of participants’ experiences rather than independent evidence that the program caused the observed change.
Contribution to personal and social development	The estimated mean change was 0.69 points, 95% CI [−2.63, 4.02], *d*_z_ = 0.07, adjusted *p* = 1.000. The interval included both negative and positive values.	Participants described program-specific experiences involving group interaction, communication, self-expression, empathy, collaboration, and listening to different perspectives.	The qualitative accounts were conceptually related to personal and social experiences, but they did not correspond exactly to the broader attitude construct assessed by the scale. The findings were therefore considered partially complementary rather than directly convergent or contradictory.
Overall reading attitude	The estimated mean increase was 7.56 points, 95% CI [2.09, 13.02], *d*_z_ = 0.47. The unadjusted *p* value was 0.008, while the Holm-adjusted *p* value was 0.057.	Participants described more deliberate reading, closer examination of texts, shared interpretations, and greater awareness of reading’s value.	The quantitative estimate and qualitative accounts were favorable in direction. However, the total-score comparison did not meet the 0.05 threshold after the Holm adjustment. The qualitative findings contextualized participants’ experiences but did not establish an overall change in reading attitude.

*Note.* CI = confidence interval. Quantitative interpretations were based on the effect estimates, confidence intervals, and Holm-adjusted *p* values reported in Table 2. The qualitative findings represent participants’ post-program accounts and should not be interpreted as independent evidence of temporal change or sustained reading behavior.

**Table 5:** Pretest–posttest comparisons of attitudes toward book-reading habits.

Dimension	Pretest M (SD)	Posttest M (SD)	Mean Change [95% CI]	t	*p*	Holm-Adjusted *p*	*d*_z_ [95% CI]
Meeting learning needs and enjoyment	75.50 (9.75)	77.00 (9.14)	1.50 [−3.61, 6.61]	0.60	0.555	1.000	0.10 [−0.23, 0.43]
Meaning and indispensability of book-reading habits	30.56 (4.03)	30.94 (4.19)	0.39 [−1.65, 2.43]	0.39	0.701	1.000	0.06 [−0.26, 0.39]
Development of book-reading habits	11.97 (2.95)	12.72 (3.09)	0.75 [−0.71, 2.21]	1.05	0.303	1.000	0.17 [−0.16, 0.50]
Overall attitudes toward book-reading habits	118.03 (15.29)	120.67 (15.20)	2.64 [−5.39, 10.67]	0.67	0.509	1.000	0.11 [−0.22, 0.44]

*Note.* (*N* = 36) for all comparisons. M = mean; SD = standard deviation; CI = confidence interval. Mean change was calculated as posttest minus pretest. The Holm adjustment was applied across the eight comparisons conducted for the two scales. Confidence intervals were not adjusted for multiple comparisons. The displayed posttest dimension means sum to 120.66 because of rounding. Their unrounded values sum to 120.6666, which is reported as 120.67 for the total score.

**Table 6:** Themes, categories, and codes derived from participants’ accounts of reading practices and experiences during the program.

Theme	Category	Codes	Coded Statements (f)
Reading routines and willingness to read during the program	Reported regular reading and time management	Reported increase in reading frequency; reported regular reading during the program; allocating time to reading; following a weekly reading schedule	26
	Willingness to read and intrinsic interest	Reporting greater willingness to read books; enjoyment of book reading; reading for pleasure; recognizing the value of reading	22
Reported strategic and purposeful reading practices	Methodological reading awareness	Using a different approach to reading; the structuring role of the role sheets; assuming a different role each week; learning how to read a book	10
	Comprehension and critical reading	Reading in depth; analyzing; reading critically; questioning; conducting research while reading	11
	Information processing and intellectual enrichment	Taking notes; interpreting symbols; expanding vocabulary; broadening one’s perspective; using imagination	14
	Pedagogical and methodological awareness	Understanding how a book can be discussed; learning how to read a book; awareness of listening and research skills; relating the experience to the teaching profession	6
Social learning and sharing	Peer discussion and sharing	Reading as a group; discussing books; encountering different ideas; sharing what was read	33
	Solidarity and communication	Group solidarity; effective communication; social interaction; developing a culture of discussion	10
	Self-expression	Expressing oneself comfortably; speaking in public; explaining one’s views	8

*Note.* Frequencies represent the number of statements coded within each category. Because a participant’s response could be coded under more than one code, the frequencies should not be interpreted as numbers of participants. These frequencies should not be used to rank the categories by their prevalence among participants or their relative importance. References to regular reading, increased reading frequency, and weekly reading routines reflect participants’ retrospective self-reports; they were not independently observed behaviors and should not be interpreted as evidence that lasting reading habits developed.

**Table 7:** Integration of quantitative and qualitative findings on attitudes toward book-reading habits.

Attitude Dimension	Quantitative Finding	Qualitative Finding with the Closest Conceptual Correspondence	Integrated Interpretation
Meeting learning needs and enjoyment	The estimated mean increase was 1.50 points, 95% CI [−3.61, 6.61], *d*_z_ = 0.10, unadjusted *p* = 0.555, and Holm-adjusted *p* = 1.000.	Participants reported learning new information, enjoying the books and group discussions, and experiencing reading as a more engaging activity.	The qualitative accounts addressed learning and enjoyment, which were conceptually related to the scale dimension. However, the quantitative estimate was small and imprecise. The accounts therefore provided information about participants’ experiences but did not establish a pretest–posttest change in this dimension.
Meaning and indispensability of book-reading habits	The estimated mean increase was 0.39 points, 95% CI [−1.65, 2.43], *d*_z_ = 0.06, unadjusted *p* = 0.701, and Holm-adjusted *p* = 1.000.	Participants described becoming more aware of the place of reading in their lives, the need to allocate time to reading, and the value of reading regularly.	The qualitative findings showed some conceptual correspondence with the meaning participants attributed to reading. Nevertheless, the quantitative estimate was close to zero and its confidence interval included values in both directions. The qualitative accounts were therefore interpreted as perceived experiences rather than evidence of change in the corresponding scale score.
Development of book-reading habits	The estimated mean increase was 0.75 points, 95% CI [−0.71, 2.21], *d*_z_ = 0.17, unadjusted *p* = 0.303, and Holm-adjusted *p* = 1.000.	Participants reported following a weekly reading schedule, allocating time to reading, using role sheets, assuming group responsibilities, and approaching books more deliberately.	The interview accounts concerned perceived routines and experiences during the program, whereas the scale dimension assessed attitudes toward developing book-reading habits. These constructs overlap only partially. The qualitative findings may help explain the reading process experienced by participants but do not demonstrate a sustained change in attitudes or reading behavior.
Overall attitudes toward book-reading habits	The estimated mean increase was 2.64 points, 95% CI [−5.39, 10.67], *d*_z_ = 0.11, unadjusted *p* = 0.509, and Holm-adjusted *p* = 1.000.	Participants described favorable experiences involving willingness to read, planned reading, critical engagement with texts, and peer sharing.	The quantitative findings did not provide clear evidence of an overall pretest–posttest change. The qualitative accounts documented how participants experienced the program but did not independently establish a change in overall attitudes toward book-reading habits.

*Note.* CI = confidence interval. Quantitative interpretations were based on the effect estimates, confidence intervals, and Holm-adjusted *p* values reported in Table 5. The qualitative findings represent participants’ post-program accounts. They should not be interpreted as direct evidence of pretest–posttest change or sustained reading behavior.

## Data Availability

The data presented in this study are available on request from the corresponding author. The data supporting the findings of this study are not publicly available because they contain information that could compromise participant privacy.

## Supplementary Materials

The following supporting information can be downloaded at: https://www.mdpi.com/article/10.3390/bs16101757/s1, Supplementary File S1: The semi-structured interview guide, including Table S1, “Original Turkish semi-structured interview guide, English translation, and conceptual basis used in question development; Supplementary File S2: Descriptions of the literature-circle roles, blank role sheets, and the blank observer form used during the program.
