# Supplementary material for: Changes in Reading Attitudes and Attitudes Toward Book-Reading Habits Following Literature Circles Among Preservice Turkish Language Teachers: An Embedded Mixed-Methods Study
Source: Behav Sci (Basel). 2026 Sep 27;16(10):1757. doi: 10.3390/bs16101757 (PMC13701446; doi:10.3390/bs16101757)
Supplement: Supplementary file 1 [file behavsci-16-01757-s001.zip › behavsci-4551851-Supplementary File S1.pdf]

Table S1. Original Turkish semi-structured interview guide, English translation, and conceptual basis used in question development.

Question number	Original Turkish wording	English translation	Conceptual basis and primary focus
1	Okuma çemberi uygulamasında yer almak, genel olarak kitap okuma eylemine bakış açınızı veya yaklaşımınızı nasıl etkiledi? Bu durumu spesifik örneklerle ve nedenleriyle açıklayınız.	How did participating in the literature circle program affect your perspective on or approach to reading books in general? Please explain this with specific examples and reasons.	Scale-informed—overall reading attitude
2	Bu süreç boyunca gerçekleştirdiğiniz okuma ve tartışma oturumları, kitaplarla kurduğunuz duygusal ve zihinsel bağı ne yönde şekillendirdi? Süreçteki hislerinizi veya düşüncelerinizi gerekçelendirerek anlatınız.	In what ways did the reading and discussion sessions conducted throughout the process shape the emotional and intellectual connection you formed with books? Please explain and provide reasons for the feelings or thoughts you experienced during the process.	Scale-informed—interest in and enjoyment of reading
3	Grup içerisindeki etkileşimlerinizin, akranlarınızla iletişim kurma, düşüncelerinizi ifade etme veya kendinizi değerlendirme biçiminize yansımaları neler oldu? Yaşadığınız somut durumlar üzerinden açıklayınız.	How were your interactions within the group reflected in the way you communicated with your peers, expressed your thoughts, or evaluated yourself? Please explain through specific situations that you experienced.	Scale-informed—contribution to personal and social development
4	Okuma çemberi deneyiminin ardından, kitap okumanın gündelik yaşamdaki veya bireysel gelişimdeki konumuna dair düşüncelerinizde bir farklılaşma oldu mu? Eğer olduysa, bu farklılaşmanın nedenlerini ve sizin için ne ifade ettiğini anlatınız.	Following the literature circle experience, did your views concerning the place of book reading in everyday life or personal development change? If so, please explain the reasons for this change and what it meant to you.	Scale-informed—perceived importance and value of reading
5	Okuma çemberi öncesi ve süreç içerisindeki okuma pratiğinizi karşılaştırdığınızda; okuma sıklığınız, kitaplara ayırdığınız zaman veya bitirdiğiniz kitap sayısı açısından neler gözlemlediniz? Bu durumu nasıl değerlendiriyorsunuz?	When comparing your reading practices before and during the literature circle program, what did you observe regarding your reading frequency, the time you devoted to reading, or the number of books you completed? How do you evaluate these observations?	Contextual question—retrospective self-reports of reading practices; not an objective behavioral measure
6	Kitaplar üzerine yapılan grup tartışmaları; yeni bilgiler edinme, farklı bakış açılarını keşfetme veya okuma sürecinden keyif alma deneyiminizi nasıl etkiledi? Somut örneklerle açıklayınız.	How did the group discussions about the books affect your experience of acquiring new information, discovering different perspectives, or enjoying the reading process? Please explain with specific examples.	Scale-informed—meeting learning needs and enjoyment
7	Bu uygulama, kitap okumayı yaşamınızda vazgeçilmez bir ihtiyaç ya da yalnızca zaman ayrılan bir tercih olarak görme biçiminizi etkiledi mi? Her iki durum için de düşüncelerinizi gerekçelendiriniz.	Did this program affect whether you regarded book reading as an indispensable need in your life or merely as an activity for which you chose to make time? Please explain your views concerning both possibilities.	Scale-informed—meaning and indispensability of book-reading habits

8	Uygulama süresince edindiğiniz deneyimlerin, düzenli kitap okuma alışkanlığı geliştirme veya bu alışkanlığı sürdürme konusundaki isteğinize etkisi oldu mu? Nedenleriyle belirtiniz.	Did the experiences you gained during the program affect your willingness to develop or maintain a regular book-reading habit? Please explain your reasons.	Scale-informed—attitudes and intentions concerning the development of book-reading habits
9	Okuma çemberi süresince üstlendiğiniz farklı roller hakkındaki genel düşünceleriniz nelerdir? Bu rollerden hangisini üstlenmek sizin için daha anlamlıydı ve neden?	What are your overall views of the different roles you undertook during the literature circle program? Which of these roles was more meaningful to you, and why?	Implementation-focused—roles undertaken by participants
10	Uygulamanın farklı aşamalarını düşündüğünüzde, sizin için en kolay ve en zorlayıcı olan kısımlar hangileriydi? Bu durumların nedenlerini açıklayınız.	When considering the different stages of the program, which parts were the easiest and the most challenging for you? Please explain the reasons for these experiences.	Implementation-focused—facilitating and challenging aspects of implementation
11	Okuma çemberi uygulamasının tüm sürecini değerlendirdiğinizde, olumlu ve olumsuz bulduğunuz yönler nelerdir? Gelecekte yapılacak benzer uygulamalar için önerilerinizi belirtiniz.	When evaluating the literature circle program as a whole, which aspects did you consider positive and negative? Please provide your recommendations for similar programs in the future.	Implementation-focused—overall evaluation, negative experiences, and recommendations
