# Supplementary material for: Changes in Reading Attitudes and Attitudes Toward Book-Reading Habits Following Literature Circles Among Preservice Turkish Language Teachers: An Embedded Mixed-Methods Study
Source: Behav Sci (Basel). 2026 Sep 27;16(10):1757. doi: 10.3390/bs16101757 (PMC13701446; doi:10.3390/bs16101757)
Supplement: Supplementary file 1 [file behavsci-16-01757-s001.zip › behavsci-4551851-Supplementary File S2.pdf]

Literature Circle Role Descriptions Blank Role Sheets and Observer Form

Purpose and language note

This file provides the role framework and blank forms used to structure the seven-week literature-circle program. The intervention and all participant-facing materials were administered in Turkish. The forms below are presented in English translation for supplementary publication, with the original Turkish role names retained in each heading. The role sheets were developed by the researcher by drawing on examples in the literature and were adapted to the procedures of the present study (Avcı et al., 2013; Doğan et al., 2019).

Role assignment in the present study

- Four roles were treated as core roles: Connector, Questioner, Passage Expert, and Illustrator.
- Five roles formed the optional-role pool: Summarizer, Word Detective, Setting Tracker, Character Expert, and Observer.
- The Observer role was required in every group in this study. In each six-member group, the four core roles and the Observer were assigned, and the sixth member selected one of the remaining optional roles.
- Roles were rotated weekly. During the seven-week program, every participant assumed each core role at least once; the remaining optional assignments reflected participant preferences.
- Participants completed their assigned role sheets for the weekly reading, used the sheets during the group discussion, and uploaded them to the Google Classroom virtual classroom after the meeting.

Overview of the literature circle roles

Role	Status in this study	Primary responsibility
Connector<br>Bağ Kurucu	Core	Identifies and explains connections between the book and everyday life, other texts, authors, genres, or works.
Questioner<br>Sorgulayıcı	Core	Prepares discussion questions and facilitates the group discussion.
Passage Expert<br>Bölüm Uzmanı	Core	Selects important or noteworthy passages and explains the reasons for each selection.
Illustrator<br>Ressam	Core	Represents a scene, symbol, expression, sentence, or passage from the book in visual form.
Summarizer<br>Özetleyici	Optional	Prepares a concise account of the book's central ideas and events.
Word Detective<br>Kelime Avcısı	Optional	Identifies unfamiliar, notable, or important words and shares their meanings.
Setting Tracker<br>Hareket İzcisi	Optional	Describes in detail the places and settings in which the events occur.
Character Expert<br>Karakter Ustası	Optional	Examines and presents the defining characteristics of the book's characters.
Observer<br>Gözlemci	**Required in every group**	Coordinates and records the meeting, documents preparation and participation, and monitors the submission of weekly materials.

General completion instructions

- Complete the sheet for the pages assigned for that week before attending the meeting.
- Bring the completed sheet to the meeting and use it when presenting your role and contributing to the discussion.
- Add brief notes during the discussion when peers' comments change, extend, or qualify your initial interpretation.
- Upload the completed role sheet to the Google Classroom virtual classroom after the meeting.

Form S2.1 Connector Role Sheet

Original Turkish role name Bağ Kurucu

Purpose Identify meaningful connections between the book and the world beyond the book, and explain why those connections matter.

**Participant name or ID**		**Group**	
**Book**		**Week**	
**Assigned pages**		**Date**	

Instructions Record connections to personal or everyday experiences, another text, an author, a genre, a work, or a broader social issue. Support each connection with a passage or page reference.

Type of connection	Passage or page	Connection identified	Why the connection matters

Points added or reconsidered during the group discussion

Form S2.2 Questioner Role Sheet

Original Turkish role name Sorgulayıcı

Purpose Prepare questions that help group members discuss, interpret, and evaluate the weekly reading.

**Participant name or ID**		**Group**	
**Book**		**Week**	
**Assigned pages**		**Date**	

Instructions Use mainly open-ended questions. Questions may address motives, relationships, causes, consequences, meanings, alternative interpretations, or links with experience. Note relevant textual evidence and key responses raised during the meeting.

No.	Discussion question	Reason for asking	Textual evidence and discussion notes

Follow-up question developed during the discussion

Form S2.3 Passage Expert Role Sheet

Original Turkish role name Bölüm Uzmanı

Purpose Select passages that are especially important or noteworthy and explain the basis for each selection.

**Participant name or ID**		**Group**	
**Book**		**Week**	
**Assigned pages**		**Date**	

Instructions A passage may be informative, surprising, humorous, upsetting, engaging, thought-provoking, confusing, or important for another reason. Record its location, explain your choice, and decide how you will introduce it to the group.

Page or opening words	Reason for selection	Why it is important	Discussion notes

Passage that generated the most discussion and why

Form S2.4 Illustrator Role Sheet

Original Turkish role name Ressam

Purpose Create a visual representation of a scene, symbol, expression, sentence, or passage from the weekly reading.

**Participant name or ID**		**Group**	
**Book**		**Week**	
**Assigned pages**		**Date**	

Instructions Select an element that is meaningful to you, record its location in the book, and represent it below. The visual may be literal or symbolic. Be prepared to explain your choices before hearing the group's interpretations.

Selected element	Page or passage	Reason for selection

Drawing or visual representation

Group interpretations and your explanation

Form S2.5 Summarizer Role Sheet

Original Turkish role name Özetleyici

Purpose Prepare a concise and coherent account of the central ideas and events in the weekly reading.

**Participant name or ID**		**Group**	
**Book**		**Week**	
**Assigned pages**		**Date**	

Instructions Distinguish essential information from minor details. Organize the summary in a logical sequence and include the central issue, major events or ideas, and the outcome or current point reached in the book.

Central issue or idea	Main characters or participants	Setting or context

Concise summary

Key turning point or most important development

Revision or addition after the group discussion

Form S2.6 Word Detective Role Sheet

Original Turkish role name Kelime Avcısı

Purpose Identify unfamiliar, surprising, notable, or important words and share their meanings with the group.

**Participant name or ID**		**Group**	
**Book**		**Week**	
**Assigned pages**		**Date**	

Instructions First consider the meaning suggested by the sentence or passage. Then verify the meaning using an appropriate source. Record enough context to show how the word is used in the book.

Page or paragraph	Word	Context or clue	Meaning in context	Source used

Word that contributed most to the discussion and why

Form S2.7 Setting Tracker Role Sheet

Original Turkish role name Hareket İzcisi

Purpose Identify and describe the places and settings in which the events occur.

**Participant name or ID**		**Group**	
**Book**		**Week**	
**Assigned pages**		**Date**	

Instructions Record the locations in the weekly reading, support them with a page or passage reference, and describe the details that help the reader understand each setting. Consider how the setting relates to the events or characters.

Setting or place	Page or evidence	Descriptive details	Events and significance

Connections or changes among the settings

Form S2.8 Character Expert Role Sheet

Original Turkish role name Karakter Ustası

Purpose Examine the defining characteristics of the main and supporting characters and explain how those characteristics are shown in the text.

**Participant name or ID**		**Group**	
**Book**		**Week**	
**Assigned pages**		**Date**	

Instructions Use actions, statements, relationships, decisions, and other textual evidence when describing a character. Distinguish your interpretation from information stated directly in the book.

Main character profile

Feature	Notes and textual evidence
Character name	
Physical characteristics	
Psychological or emotional characteristics	
Role in the text	
Problems or conflicts encountered	
Achievements, decisions, or important actions	
Positive characteristics	
Negative characteristics	

Form S2.8 Character Expert Role Sheet Continued

Use this page to compare the supporting characters and record evidence from the text.

Supporting characters

Character	Key characteristics	Role or relationship	Evidence

Connections or contrasts among the characters

Form S2.9 Observer Form

Original Turkish role name Gözlemci

Purpose Coordinate and document the weekly meeting, observe preparation and participation, and monitor the submission of the required materials.

Instructions Confirm the meeting date and time, arrange the session recording, complete the tables below during or immediately after the meeting, and upload the observer form with the group's weekly materials.

**Group**		**Week**	
**Book**		**Date**	
**Location**		**Meeting duration**	

Attendance and role allocation

Participant name or ID	Assigned role	Present	Role sheet prepared	Upload confirmed	Notes

Attendance or role allocation notes

Form S2.9 Observer Form Continued

Meeting documentation

Complete the checklist for the meeting as a whole and record any departure from the planned procedure.

Meeting documentation checklist

Item	Yes	No	Partly	Notes
The date and time were communicated to the group.				
The meeting was held and the discussion was completed.				
A video excerpt of approximately 10 to 15 minutes was recorded.				
All available role sheets were used during the discussion.				
The weekly poster was completed.				
The role sheets, poster, and observer form were prepared for upload.				

Unexpected events or departures from the planned procedure and action taken

Follow-up actions and upload notes

Form S2.9 Observer Form Continued

Participant and role evaluation

Complete one copy of this checklist for each group member, or duplicate the table as needed. Base the ratings on preparation for the assigned role and participation during the meeting.

**Participant name or ID**		**Role evaluated**	
**Observer name or ID**		**Date**	

Criterion	Yes	No	Partly	Evidence or comment
Participated in the group meeting.				
Prepared the assigned role sheet.				
Presented the assigned role effectively.				
Communicated respectfully and clearly within the group.				
Participated actively in the group discussion.				
Shared ideas and contributed to the content of the discussion.				
Responded to questions when appropriate.				
Listened attentively to other group members.				
Contributed to the preparation of the weekly poster.				
Completed any responsibilities carried over from the previous meeting.				

Overall observation

References for the development of the role materials

Avcı, S., Baysal, N., Gül, M., & Yüksel, A. (2013). The effect of literature circles on reading comprehension skills. *Journal of Theoretical Educational Science*, *6*(4), 535–550. https://doi.org/10.5578/keg.6710

Doğan, B., Çermik, H., Ateş, S., & Yıldırım, K. (2019). Öğretmen adaylarının okuma çemberlerinde üstlendikleri rollere ilişkin görüşleri [Preservice teachers' views on the roles they assumed in literature circles]. *Ana Dili Eğitimi Dergisi*, *7*(1), 141–157. https://doi.org/10.16916/aded.482428
